# Nuclear derivatives and axonal projections originating from rhombomere 4 in the mouse hindbrain

**DOI:** 10.1007/s00429-017-1416-0

**Published:** 2017-05-03

**Authors:** Maria Di Bonito, Michèle Studer, Luis Puelles

**Affiliations:** 1 Université Côte d’Azur, CNRS, Inserm, iBV, Institut de Biologie Valrose, 06108 Nice, France; 2 Department of Human Anatomy and Psychobiology, Faculty of Medicine, University of Murcia, 30071 Murcia, Spain; 3 Instituto Murciano de Investigación Biosanitaria (IMIB-Arrixaca), LAIB, Avda Buenavista s/n, El Palmar, 30120 Murcia, Spain

**Keywords:** Fate map, Rhombomere 4, *b1r4-Cre* line, Sensorimotor systems

## Abstract

The r4-derived territory is located in the pontine region of the brainstem, forming a wedge-shaped slice that broadens from the choroidal roof to the ventral midline. R4-derived neuronal populations migrate radially inside and tangentially outside this rhombomere, forming nuclei of the sensorimotor auditory, vestibular, trigeminal and reticular systems. R4-derived fibre tracts contribute to the lateral lemniscus, the trigeminothalamic tracts, the medial tegmental tract and the medial forebrain bundle, which variously project to the midbrain, thalamus, hypothalamus and telencephalon. Other tracts such as the trigeminocerebellar and vestibulocerebellar tracts reach the cerebellum, while the medial and lateral vestibulospinal tracts, and the reticulospinal and trigeminal oro-spinal tracts extend into the spinal cord. Many r4-derived fibres are crossed; they decussate to the contralateral side traversing the midline through the cerebellar, collicular and intercollicular commissures, as well as the supraoptic decussation. Moreover, some fibres enter into the posterior and anterior commissures and some terminals reach the septum. Overall, this study provides an overview of all r4 neuronal populations and axonal tracts from their embryonic origin to the adult final location and target.

## Introduction

Hindbrain neurons process and relay sensory information, control vital functions and contribute to motor coordination. The complex circuitry formed by sensory, reticular/interneuronal and motor neurons is established during development and depends on a spatially and temporally ordered sequence of neuronal specification, cell migration and axonal pathfinding.

The immature rostral hindbrain of vertebrates is overtly segmented into molecularly and morphologically distinct developmental (histogenetic) compartments, called rhombomeres (r), forming the series r1–r6 along the anteroposterior (AP) axis (Fraser et al. 1990; Graham et al. 2014; Kiecker and Lumsden 2005; Lumsden 1990; Puelles et al. 2013; Vaage 1969). Each of these rhombomeres, except r1, expresses a particular combination of *Hox* and other genes, which specify their respective molecular identity and developmental fate (Krumlauf et al. 1993; Tomas-Roca et al. 2016; Tumpel et al. 2009). The paralogue *Hox* groups 1–3 operate instructively across this domain in an anteroposterior series that follows the principle of 3′–5′ collinearity (Krumlauf et al. 1993; Parrish et al. 2009). The caudal medullary hindbrain has instead a hidden rhombomeric organization, which is molecularly detectable using *Hox* gene markers, but is not distinct morphologically as visible transverse bulges. This domain was divided into five cryptorhombomeres (r7–r11) (Cambronero and Puelles 2000; Puelles et al. 2013), which also display typical anteroposterior (3′–5′) spatial colinearity of *Hox* gene expression; the rostral expression limits of the paralogue *Hox* groups 4–8 sequentially coincide with the experimentally fate-mapped limits of r7–r11 (Marin et al. 2008; Tomas-Roca et al. 2016). A similar case is posed by the isthmic cryptorhombomere (r0), which only can be delimited molecularly from r1 (Aroca and Puelles 2005; Puelles et al. 2013). Embryonic hindbrain neuromeres or segments give rise to the prepontine region (isthmus plus r1 and r2; includes the cerebellum), the pons (r3, r4), the retropontine region (r5, r6) and the medulla oblongata (r7–r11) in the adult brain (Puelles 2013; Puelles et al. 2013). The cryptically delimited isthmus (r0) and r1 are patterned under the influence of the isthmic organizer, which also induces across their dorsal aspect the cerebellum.

Long-term fate-mapping studies of rhombomeres in chicken (Cambronero and Puelles 2000; Cramer et al. 2000; Diaz et al. 1998; Marin et al. 2008; Marin and Puelles 1995; Puelles et al. 2013; Tan and Le Douarin 1991) and mouse (Di Bonito et al. 2013a, 2015; Di Meglio et al. 2013; Farago et al. 2006; Gray 2013; Oury et al. 2006; Pasqualetti et al. 2007; Tomas-Roca et al. 2016) have revealed that various rhombomeres contribute to neurons of the auditory, vestibular, trigeminal, somatosensory, reticular and precerebellar systems.

Within each rhombomere, inductive signals and specific transcriptional pathways confer a positional pattern on the neural progenitors, linking their position along the AP and DV axes to differential specification of characteristic neuron subtypes, specific migratory behaviour of given cell populations and axonal projections to characteristic targets, contributing to the complexity of hindbrain sensorimotor circuits (reviews by Di Bonito et al. 2013b; Philippidou and Dasen 2013). Neuromeric classification of classic anatomic entities in the hindbrain often entails the recognition of their bi- or plurineuromeric composition (e.g. postmigratory basilar pons placed across r3 and r4, or trigeminal motor nucleus originated across r2 and r3). Moreover, the interaction between the unique molecular identity of each rhombomere and a shared mechanism of dorsoventral patterning (dorsalization versus ventralization) leads to functionally characteristic alar and basal derivatives of each rhombomere and cryptorhombomere, which participate in the plurineuromeric modular neuronal arrangements obtained within hindbrain columns (e.g. the cochlear, vestibular and trigeminal sensory columns).

In the mouse, the organization of the facial somatosensory map is related to the r2 and r3 components of the principal trigeminal sensory nucleus (Oury et al. 2006; Pouchelon et al. 2012). Vestibular nuclei originate from multiple rhombomeres ranging from r2 to r9, and the corresponding segmental modules display specific axonal projections to distinct targets (Chen et al. 2012; Di Bonito et al. 2015; Pasqualetti et al. 2007). Some neuromere-selective projection patterns have been described. For instance, the chicken r4 vestibular module does not produce vestibulo-ocular projection neurons and only few of them arise in mice r4, while r4 contributes mainly contralaterally and ipsilaterally projecting vestibulospinal neurons (Di Bonito et al. 2015; Diaz et al. 1998, 2003).

In the auditory system, the cochlear nuclear complex represents an important plurineuromeric relay station of the auditory pathway, which derives from the dorsal rim of the r2–r5 units, in particular from *Atoh1-* and *Ptf1*-expressing progenitor domains (Farago et al. 2006; Fujiyama et al. 2009; Maricich et al. 2009; Rose et al. 2009). R5 contributes massively to the superior olivary complex (SOC) (Maricich et al. 2009), whereas r4 contributes only partially to the LSO (Di Bonito et al. 2013a; Marin and Puelles 1995). Along the DV axis, the SOC derives from both rhombic lip and non-rhombic lip progenitor domains (Altieri et al. 2015; Machold and Fishell 2005; Marrs et al. 2013), namely from *Atoh1*- (Maricich et al. 2009), *Wnt1*- (Fu et al. 2011; Marrs et al. 2013) and *Wnt3a*-positive (Louvi et al. 2007) rhombic lip domains dorsally and from an *En1*-positive lineage (Altieri et al. 2015; Marrs et al. 2013) ventrally, respectively. The ventral nucleus of the lateral lemniscus (VLL) derives from r4 (Di Bonito et al. 2013a), while the dorsal nucleus of the lateral lemniscal (DLL) derives from r1 (as is suggested by its *Pax7* expression, which only occurs in the r1 mantle; Lorente-Canovas et al. 2012; Moreno-Bravo et al. 2014 misidentified it as ILL; Allen Developing Mouse Brain Atlas).

Different rhombomeres thus contribute to different functional modular subsystems of the auditory system. R4 contributes mainly to sound perception; moreover, r4-derived cochlear sensory neurons contribute jointly with basal plate-derived motor structures to the two distinct auditory sensorimotor feedback subcircuits that protect the cochlea from acoustic overstimulation; r2, r3 and r5 instead contribute to sound localization circuitry (Di Bonito et al. 2013a, b).

Precerebellar nuclei, essential for the relay of peripheral proprioceptive signals and cortical collateral copy input to the cerebellum, are generated from the rhombic lip in the caudal hindbrain (precerebellar lip), which extends from r6 to r11 (Cambronero and Puelles 2000; Hidalgo-Sanchez et al. 2012; Landsberg et al. 2005; Machold and Fishell 2005; Marin and Puelles 1995; Puelles et al. 2013; Rodriguez and Dymecki 2000; Wang et al. 2005; Wingate 2001). They originate along the dorsoventral axis from discrete molecularly defined pools of progenitor cells expressing *Atoh1, Ngn1, Mash1* and *Ptf1a* (reviewed by Ray and Dymecki 2009). In particular, the basilar pontine nuclei derive from the lower rhombic lip over r6–r8. They follow a subpial tangential migration path into the basilar region of r3 and r4, during which neuronal subsets maintain their relative positions (Di Meglio et al. 2013).

Finally, r4 is known to produce in its basal plate the facial branchiomotor neurons (FBM) as well as inner ear efferent neurons (IEE) whose axons course through the facial (7n) and vestibulocochlear (8n) cranial nerves (Auclair et al. 1996; Bruce et al. 1997; Simon and Lumsden 1993).

As is well known, the *Hoxb1* gene specifies singularly the identity of the dorsoventral sets of progenitor fields found within r4 (Di Bonito et al. 2013a, 2015; Studer et al. 1996). A late r4-*Hoxb1* enhancer specifically maintains *Hoxb1* transcription at high levels in the whole r4 (Studer et al. 1998, 1994); later in development *Hoxb1* expression becomes restricted to distinct dorsoventral neuronal subdomains corresponding to specific sensory and motor columns (Gaufo et al. 2000; Gavalas et al. 2003). *Hoxb1-*deficient mouse mutants and human patients carrying mutations in the *HOXB1* gene have severe impairments in the auditory, trigeminal and vestibular systems (Chen et al. 2012; Di Bonito et al. 2013a, 2015; Studer et al. 1996; Webb et al. 2012). *Hoxb1* activates an r4-specific developmental programme and represses locally the emergence of r3-like molecular and cellular features by inhibiting *Hoxa2, Atoh1* and *Ascl1* expression in the trigeminal, auditory and vestibular columns via different molecular pathways (Chen et al. 2012; Di Bonito et al. 2013a, b, 2015; Gaufo et al. 2000). As a consequence, lack of function of *Hoxb1*, the major r4 selector gene, entails that r4-derived motor and sensory neurons of the auditory, vestibular and trigeminal systems acquire molecular profiles and neuronal behaviours (migration and axonal pathfinding) characteristic of their r3 analogs. Thus, repatterning related to *Hoxb1* loss-of-function changes the molecular identity of r4 progenitors into an r3-like identity and promotes a complete reorganization of several crucial axonal pathways and neuronal circuits in the mature brainstem.

In mouse, the use of the *Cre–lox* system and of various *Cre-recombinase* lines, either specific to distinct rhombomeres (Altieri et al. 2015; Di Bonito et al. 2013a, 2015; Di Meglio et al. 2013; Farago et al. 2006; Jensen et al. 2008; Maricich et al. 2009; Oury et al. 2006; Pasqualetti et al. 2007), or to specific DV subdomains (Altieri et al. 2016; Fujiyama et al. 2009; Kim et al. 2008; Marrs et al. 2013; Rose et al. 2009; Storm et al. 2009; Wang et al. 2005), have been instrumental in mapping the origin of some hindbrain neuronal subtypes, eventually following their migratory displacements during development and their projections. However, only few rhombomere-specific lines including *Rse-Cre* (r2) (Awatramani et al. 2003), *Krox20* ^*Cre*^ or *Egr2* ^*Cre*^ (r3/r5) (Voiculescu et al. 2000), *Hoxb1* ^*Cre*^ (r4 to posterior) (Arenkiel et al. 2003) and *Hoxa3-Cre* (r5/r6) (Di Meglio et al. 2013) have been used to dissect the assembly of sensorimotor systems.

In order to study the neuronal subtypes originating from r4, we generated an r4-restricted *Cre* driver in the mouse (named *b1r4-Cre*) (Di Bonito et al. 2013a). The *b1r4-Cre* is a transgenic mouse line that has been obtained using a well-characterized r4 enhancer from the *Hoxb1* locus (Studer et al. 1994) to express the *Cre*-recombinase gene exclusively in r4. This contrasts with the previous lines *Hoxb1* ^*Cre*^ (Altieri et al. 2016; Arenkiel et al. 2003; Farago et al. 2006; Maricich et al. 2009; Marrs et al. 2013) and *r4-Cre* (Geisen et al. 2008; Oury et al. 2006) which were described as r4-specific lines, though they invariably also display additional *Cre* expression caudal to r4. In the CNS, no ectopic *Cre* expression has been observed in the transgenic *b1r4-Cre* so far (Di Bonito et al. 2013a; their Figure S1D, and data not shown), but the theoretic possibility of some ectopic expression cannot be fully discounted. Crossing of the *b1r4-Cre* line with the *ROSA26-YFP* reporter line has allowed us to fate map selectively all r4 derivatives from E9.0 until adulthood. Previously, we described summarily the auditory, trigeminal and vestibular r4 derivatives (Di Bonito et al. 2013a, b, 2015). In this paper, we provide a full overview at different stages of all r4 neuronal populations and axonal tracts, followed from their early embryonic origins to the final adult locations and targets.

## Materials and methods

### Animals

The *b1r4-Cre* mice (Di Bonito et al. 2013a) were crossed with *ROSA26-YFP* reporter mouse (Srinivas et al. 2001) to obtain double heterozygous *b1r4-Cre*/*YFP* progeny, in which r4 and r4 derivatives are permanently labelled. All mouse experiments were performed according to the protocols approved by the Institutional Animal Care and Use Committee of the University of Nice Sophia Antipolis, Nice, France.

### Tissue preparation

Adult and P8 mice were perfused with cold 4% paraformaldehyde (PFA) in phosphate-buffered saline (PBS) pH 7.4. Embryos and perfused brains were fixed overnight in buffered 4% PFA. Tissues were cryoprotected with 10, 20 and 30% sucrose in PBS and frozen in OCT embedding matrix (Kaltek), to be cryostat-sectioned at 16 µm thickness (E10.5, E11.5 and E12.5 embryos, sagittal plane; E14.5 embryos, sagittal and r4-adapted horizontal and coronal section planes; E16.5 brains, sagittally and horizontally, and E18.5 brains, sagittally, as well as in r4-adapted horizontal and coronal section planes). P8 and adult brains were cut 20 µm thick sagittally or coronally, respectively.

### Immunohistochemistry and Nissl staining

Immunohistochemistry was performed as previously described (Di Bonito et al. 2013a), using rabbit polyclonal anti-GFP antibody (1:500, Molecular Probes) as a primary antibody, which cross-reacts with the YFP protein. Nissl staining of cryostat sections was performed using standard procedures and the slides were mounted with EUKITT mounting medium.

### Immunofluorescence

Sagittal and coronal brain cryosections were incubated for 1 h at room temperature with blocking solution (10% goat serum; 0.3% Triton X-100 in PBS) and then overnight at 4 °C with primary antibodies diluted in the hybridization buffer (3% goat serum; 0.3% Triton X-100 in PBS): rabbit anti-GFP (1:500, Molecular Probes) and mouse anti-Pax7 (1:100, Developmental Studies Hybridoma Bank). Tissue was washed in PBS and incubated for 1 h at room temperature with secondary antibodies: Alexa Fluor 488 (green) goat anti-rabbit and Alexa Fluor 555 (red) goat anti-mouse (1:400, Molecular Probes). The slides were mounted with 2% *N*-propyl gallate in 90% glycerol in PBS (Sigma P3130).

### *In situ* hybridization

*In situ* hybridization for *Atoh7, vGluT2, Gad67* and *Gata3* was performed as previously described (Di Bonito et al. 2013a).

### Image analysis

Digital bright-field microphotographs were taken on a Leica DM 6000B microscope equipped with a Leica DFC310 FX colour camera, and processed with Adobe Photoshop CS5 software using the Photomerge function to obtain a panorama of each brain section. Digital microphotographs of immunofluorescence images were acquired by laser scanning confocal microscopy using a Zeiss LSM 710 confocal microscope and converted by ZEN software. The figures were mounted and labelled using Adobe Photoshop CS5.

## Results

The r4-derived domain forms an intensely labelled wedge in the pontine region that expands from the choroidal roof down to the midline raphe. As the development proceeds, the domain becomes more and more compressed anteroposteriorly, particularly at the choroidal roof, rhombic lip area and neighbouring dorsalmost alar plate (cochlear complex).

Our description below of the diverse derivatives of r4 will be divided into those which migrate out from this rhombomere into the neighbouring ones (tangentially migrated derivatives) and those which remain inside the r4 domain (radially migrated derivatives). In addition, we will consider the labelled fibre tracts in the last section.

Radial migration refers to ventriculo-pial displacement of newborn postmitotic cells within a subregion of the neural tube wall. Radially migrated cells are stabilized permanently in the mantle close to their ventricular matrix site, building the local radial histogenetic domain. In contrast, tangentially migrating postmitotic neurons may secondarily move away from their source, invading other parts of the neural wall, either of the same or a different neuromere, at close or longer range. It is widely thought that causal mechanisms, molecular guidance and even cytoskeletal translocation mechanisms for these two types of cell migration are substantially different. Accurate description of the histogenetic processes occurring within a developmental unit such as a neuromere requires tracking any cases of tangential migration, either entering or sorting out of the unit.

### Tangentially migrated r4 derivatives

Early on, two major streams of migratory cells leave r4 and invade adjacent regions.

#### Facial motor nucleus

**Fig. 1:**
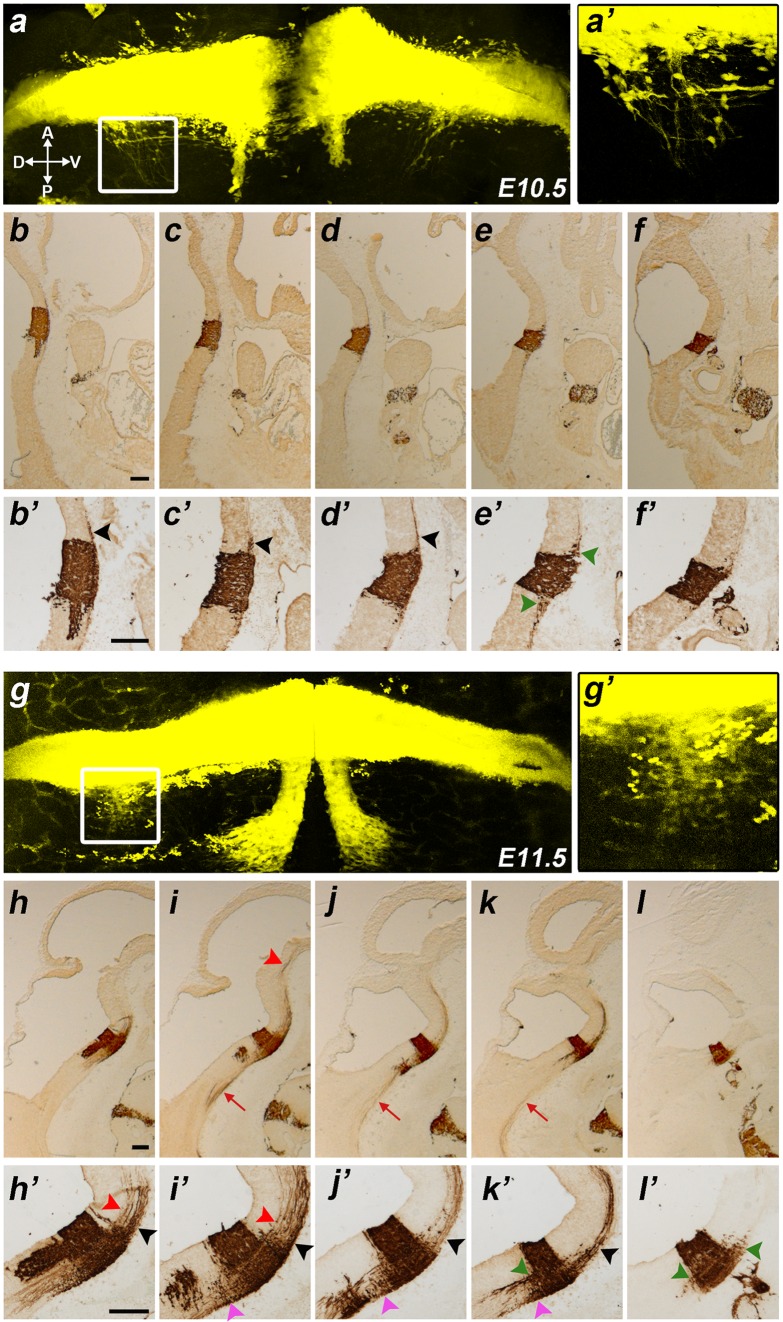
Results at **E10.5, E11.5. a, g** Hindbrain flat mounts of *b1r4-Cre*/*YFP* double heterozygote embryos at E10.5 (**a**) and E11.5 (**g**). YFP labels rhombomere 4 and r4-derived facial branchiomotor and vestibular neurons. The *boxes* in **a** and **g** indicate the areas shown at high magnification in **aʹ** and **gʹ** respectively, illustrating the estimated vestibular neurons invading r5. **b–f** and **h–l** Sagittally sectioned *b1r4-Cre*/*YFP* specimens at E10.5 (**b–f**) and E11.5 (**h–l**); each series is illustrated from medial to lateral levels and was immunoreacted with an antibody against GFP that also recognizes YFP. **bʹ–fʹ** and **hʹ–lʹ** Corresponding high-magnification views of rhombomere 4. The facial motor neurons migrate caudally through r5 next to the floor plate and turn laterally within r6 until reaching the alar pial surface where they form the facial nucleus (**a, b**/**bʹ, g, h**/**hʹ**–**j**/**jʹ**). The lateral lemniscus tract emerges rostrally from the basal part of r4 and courses obliquely lateralwards and dorsalwards to reach the prepontine alar plate (**b**/**bʹ**–**d**/**dʹ, h**/**hʹ**–**k**/**kʹ**, *black arrowheads*). The medial tegmental tract comes out of r4 and crosses the medial tegmentum of the *upper* hindbrain and reaches the isthmus (**h**/**hʹ, i**/**iʹ**, *red arrowheads*). R4-derived vestibular neurons (*dark green arrowheads*) invade r3 (**e**/**eʹ, l**/**lʹ**) and r5 (**e**/**eʹ, k**/**kʹ, l**/**lʹ**). YFP-positive reticular neurons migrate into r5 (**i**/**iʹ**–**k**/**kʹ**, *pink arrowheads*). Other fibres exit the medial basal part of r4 and descend into the spinal cord (**i–k**, *red arrow*). For abbreviations see “list of abbreviations”. *Scale bar* 200 µm

**Fig. 2:**
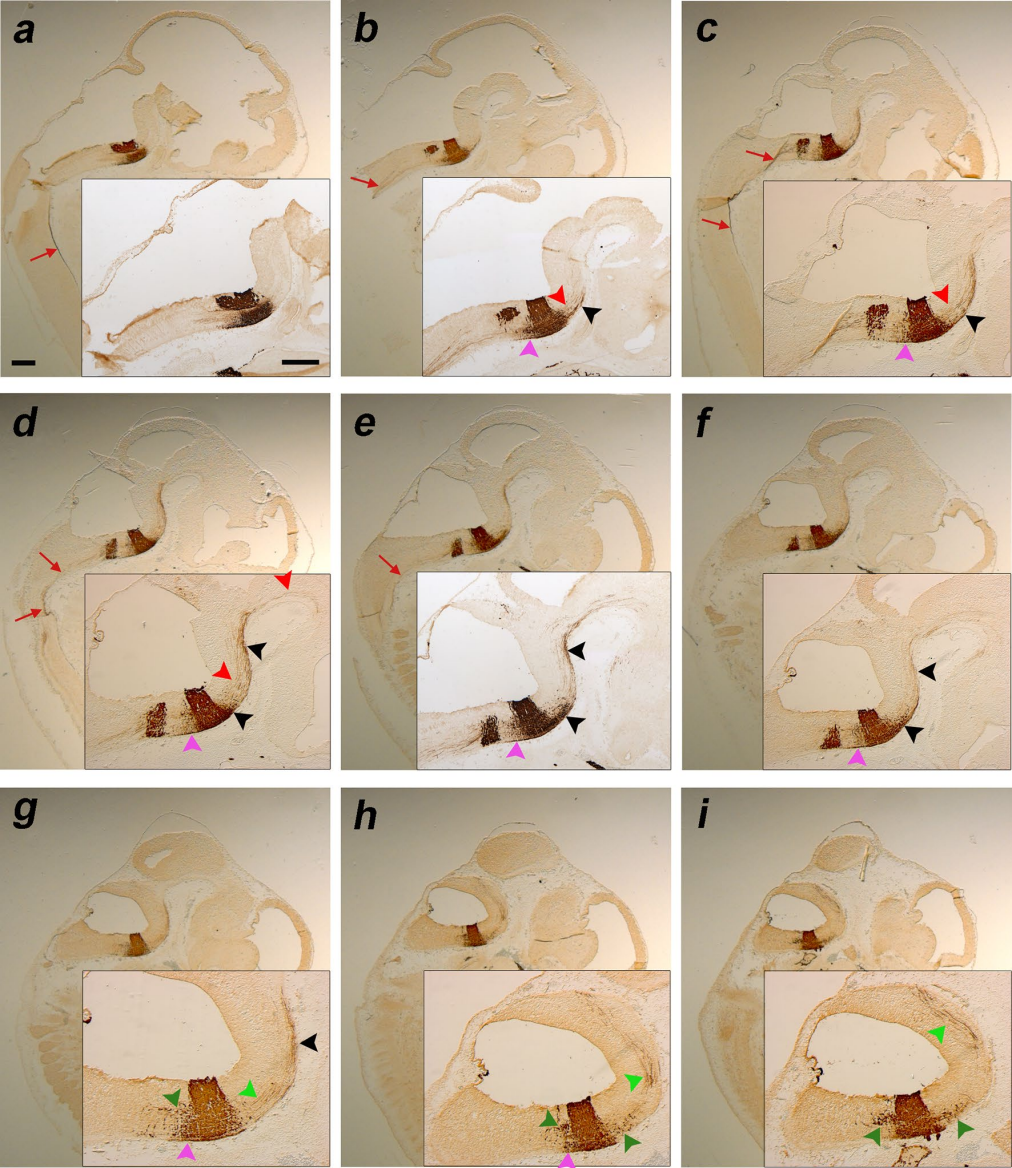
**a–i** Sagittal sections of an E12.5 *b1r4-Cre*/*YFP* double heterozygote embryo, ordered from medial to lateral, and immunostained with anti-GFP antibody; the *insets* show the corresponding higher magnification of r4 and r4 derivatives, including nuclei and fibres. The migrating facial branchiomotor neurons forming the facial knee are shown (**a**–**f**). YFP-positive neurons invading the lateral lemniscus emerge rostrally from the r4 basal longitudinal zone (**d**–**f**, *black arrowheads*) and move along this tract into the rostral prepontine hindbrain (**b**–**g**, *black arrowheads*). The labelled medial tegmental tract also extends into prepontine brainstem levels in a deeper position (**b–d**, *red arrowheads*). Other fibres exit the medial basal part of r4 and descend into the spinal cord (**a–e**, *red arrows*). Thicker fibres form laterally the trigeminal cerebellopetal tract (**g**–**i**, *light green arrowheads*). R4-derived vestibular neurons (*dark green arrowheads*) invade r3 (**h, i**) and r5 (**g**–**i**). Reticular labelled neurons are dispersed in r5, close to r4 (**b**–**h**, *pink arrowheads*). For abbreviations see “list of abbreviations”. *Scale bar* 400 µm

**Fig. 3:**
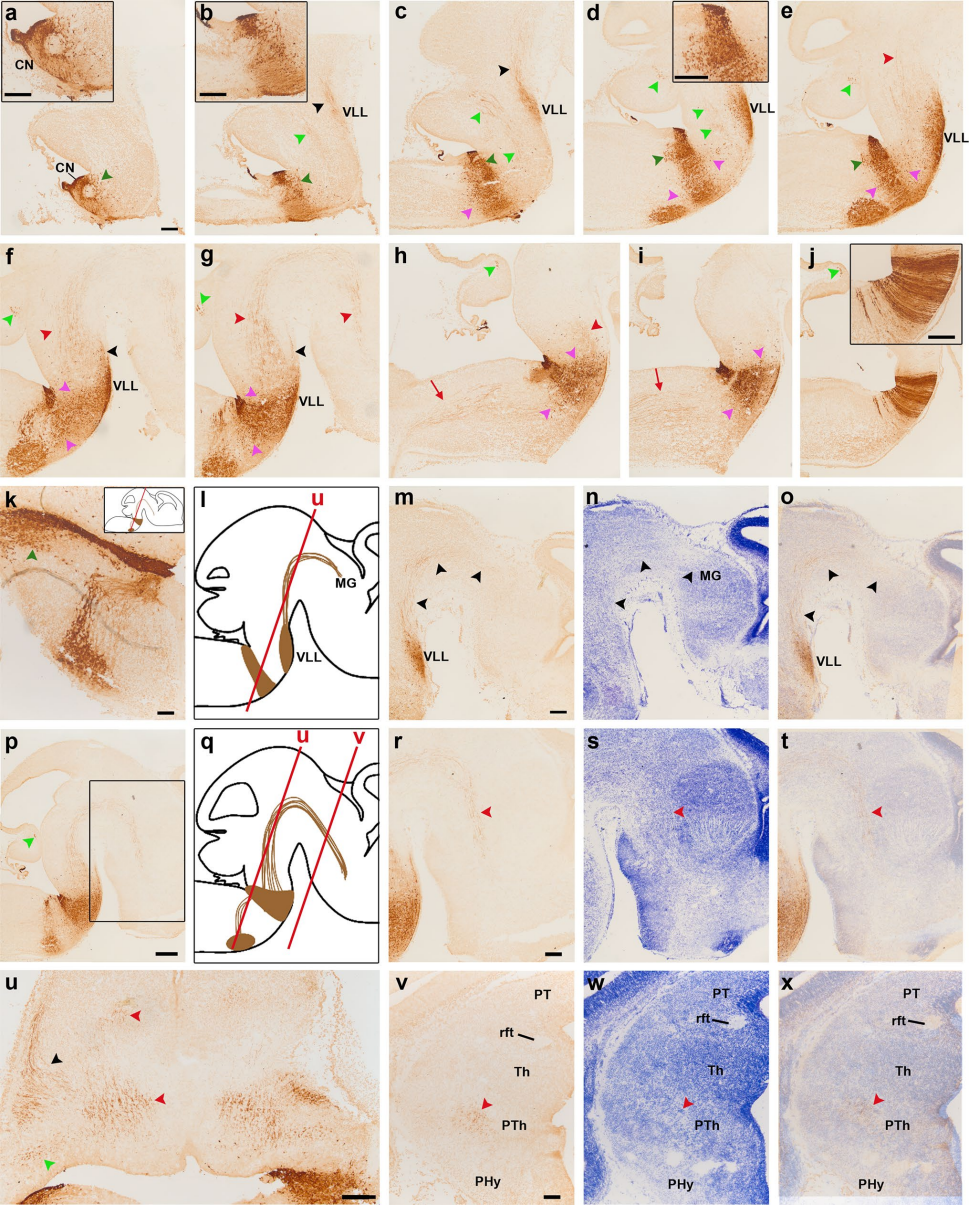
**a–j** Sagittal sections of E14.5 *b1r4-Cre*/*YFP* double heterozygote brains immunostained with anti-GFP antibody, ordered from lateral to medial, and showing the lateral lemniscus (*black arrowheads*; **b, c, f, g**), the trigeminal cerebellopetal (*light green arrowheads*; **b–h, j**), the medial tegmental (*red arrowheads*; **e–h**) and the medial spinopetal (*red arrows*; **h, i**) tracts. *Insets* show higher magnification of the cochlear nuclei (CN; **a, b**), vestibular nucleus (**d**) and median labelling of characteristic radial glia at the *midline* raphe of rhombomere 4 (**j**). The r4-derived facial neurons have migrated caudalwards from r4- into r6-derived territory, where they move first from medial to lateral, enter the alar plate and then form there superficially the facial nucleus (superficial rounded labelled mass found caudal to r4; note labelled facial nerve fibres arching from the nucleus into the facial nerve knee; **d**–**g**). Before this migration occurs, the facial axons already exited the hindbrain through r4; they thus become stretched by the caudalward migration to form the facial genu around the (unlabelled) abducens nucleus in r5 (**f, g, k, p**). R4-derived neurons also appear migrated rostrally along the lateral lemniscus tract which targets the inferior colliculus in the midbrain; this population finally stretches interstitially to the tract from r4 to the boundary between r2 and r1, forming the ventral nucleus of the lateral lemniscus complex (VLL; **b**–**g**). Labelled reticular neurons are observed to be dispersed in r5, close to r4 (**c**–**i**, *pink arrowheads*), as well as rostral to r4 (**d**–**i**, *pink arrowheads*). R4-derived vestibular neurons (*dark green arrowheads*) have invaded r5 (**d, e, k**) and r3 (**a, b**) apparently within the developing superior and inferior vestibular nuclei, respectively. **k** Roughly horizontal section relative to r4 and r6 showing the descending limb of the facial knee and the arrival of facial motoneurons at the r6 superficial stratum. The *red line* in the *inset* schema indicates the section plane. **l, q** Sagittal schemata showing our interpretation of lemniscal and facial cell populations that exit r4, as well as the labelled lateral lemniscus and medial tegmental tract fibres that grow through the midbrain into the diencephalon, as seen in non-adjacent lateral and medial sagittal sections (**m, n**) and (**r, s**), respectively, stained alternatively with anti-GFP antibody (**m, r**), or Nissl staining (**n, s**). **o, t** YFP/Nissl pairs of images (**m, n**) and (**r, s**) merged. The *box* in **p** indicates the area illustrated in sections (**r**–**t**). The YFP-positive fibres of the lateral lemniscus tract project superficially through the brachium of the inferior colliculus towards the medial geniculate nucleus and other thalamic areas (**m**–**o**, *black arrowheads*), while the deep medial tegmental tract extends rostralwards through the midbrain and diencephalic tegmentum, reaching the basal hypothalamus (**r**–**t**, *red arrowheads*). The *red lines* in the sagittal schemata in **l** and **q** indicate the section plane used in **u** and **v**–**x**; this is roughly horizontal with respect to the hindbrain, but represents a coronal section plane at the forebrain, due to the visible cephalic flexure. The lateral lemniscus (*black arrowhead*), the medial tegmental tract (*red arrowheads* in prepontine and midbrain regions; note that the tract is curved along the cephalic flexure) and the trigeminal cerebellopetal tract (*light green arrowhead*) are indicated in a horizontally sectioned hindbrain in **u**. Adjacent horizontal sections through diencephalon and hypothalamus stained with anti-GFP (**v**) and Nissl (**w**), as well as the merged YFP and Nissl images (**x**), show the fibres of the medial tegmental tract approaching the peduncular domain of the basal hypothalamus. For abbreviations see “list of abbreviations”. *Scale bar* 200 µm

Caudally, the well-known caudalward medial migratory stream of the facial motor nucleus starts to exit r4 at E10 (Fig. 1a, b/bʹ) and enters r6 at E11.5 (Fig. 1g, h/hʹ). At the latter stage, the massively labelled stream extends caudalwards in a slightly diverging course just outside of the r5 paramedian basal ventricular zone, adjacent to the floor plate and then turns laterally as it reaches the r6 basal mantle (Fig. 1g, h/hʹ–j/jʹ). At E12.5, the stream still appears connected rostrally to r4 (Fig. 2a), but already proceeds lateralwards (ventrodorsally) within r6, reaching a locus judged to correspond to the ventral rim of the r6 alar plate (this is consistent with the chick results reported by Ju et al. 2004, as well as with unpublished observations of LP in mouse). The facial nucleus primordium next spreads out progressively as the cells stream ventrolaterally, approaching the pial surface (Fig. 2b–f). In this migratory process, the motoneurons initially arch medially around the abducens motor nucleus in r5, forming the prospective genu of the facial nerve (Fig. 3k). A much smaller but distinct group of labelled neurons appears associated to the periventricular facial genu; these may represent the efferent vestibular neurons described in the literature. At higher magnification, the facial nerve axons are distinctly labelled and can be followed from the migrated facial nucleus (r6) into the genu (r5), and from there across r4 to their exit into the facial nerve root.

#### Lateral lemniscus complex

**Fig. 4:**
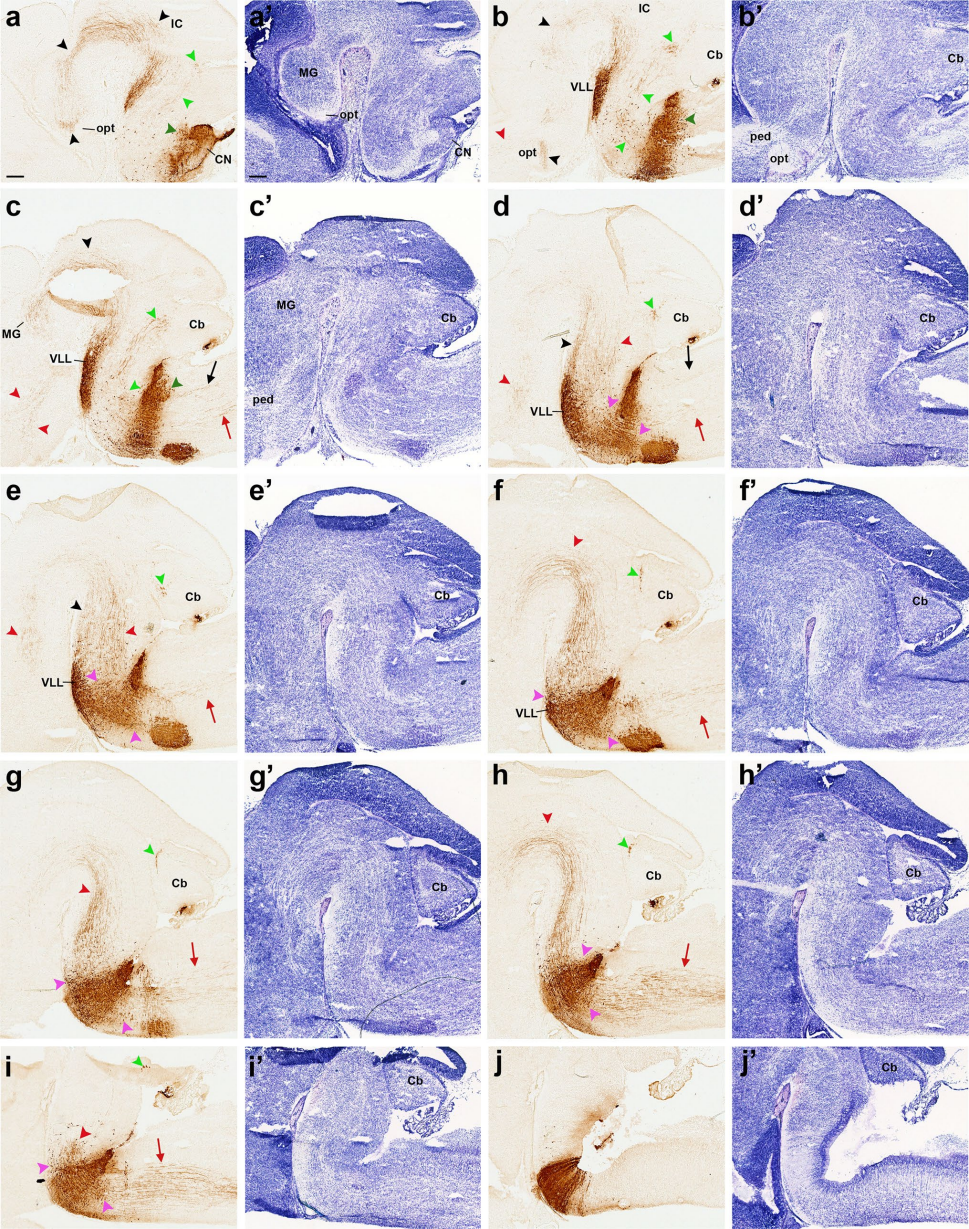
**a–jʹ** Adjacent sagittal sections of an E16.5 *b1r4-Cre*/*YFP* brain ordered from lateral to medial, and immunostained with anti-GFP antibody (**a–j**) and Nissl staining (**aʹ–jʹ**). Labelled fibres originating in the ventral nucleus of the lateral lemniscus (VLL) and fibres presumably coming from r4 projection neurons of the contralateral cochlear nuclei form the lateral lemniscus tract (*black arrowheads*), which starts to penetrate the inferior colliculus at this stage (IC; **a, b**). Many collateral fibres of this tract also extend through the brachium of the inferior colliculus (across midbrain and pretectum) into the medial geniculate nucleus of the thalamus (MG; **a**/**aʹ**–**c**/**cʹ**). Rostral to the medial geniculate, these lateral lemniscal fibres adopt a subpial position ventral to the optic tract and extend into the supraoptic decussation (opt; **a**/**aʹ**, **b**/**bʹ**; *black arrowheads*). A thick packet of YFP-positive fibres, the medial tegmental tract (*red arrowheads*), comes out of r4 medially (**i**/**iʹ**; *red arrowhead*), crosses longitudinally the medial tegmentum of the prepontine hindbrain, the midbrain and the diencephalon (**d–h**; *red arrowheads*), and also courses longitudinally within the basal hypothalamus (**c**/**cʹ**; *red arrowheads*); there, some fibres turn sharply dorsalwards into the medial forebrain bundle, thus reaching the telencephalon (ped; **b**/**bʹ, c**/**cʹ**; *red arrowheads*). YFP-labelled fibres, which seem to stream out of the trigeminal sensory column at the level of subnucleus oralis, reach the cerebellum and partly cross the cerebellar commissure, forming the trigeminal cerebellopetal tract (**a**–**i**, *light green arrowheads*). Medial spinopetal (**c–i**, *red arrows*) and lateral vestibulospinal (**c, d**, *black arrows*) tracts are labelled, jointly with YFP^+^ (r4-derived) cochlear nuclei portions (CN; **a**), vestibular neurons (*dark green arrowheads*) invading r3 (**a**) and r5 (**c**), and reticular neurons dispersed in r2, r3 and r5 (**d–i**, *pink arrowheads*). For abbreviations see “list of abbreviations”. *Scale bar* 200 µm

**Fig. 5:**
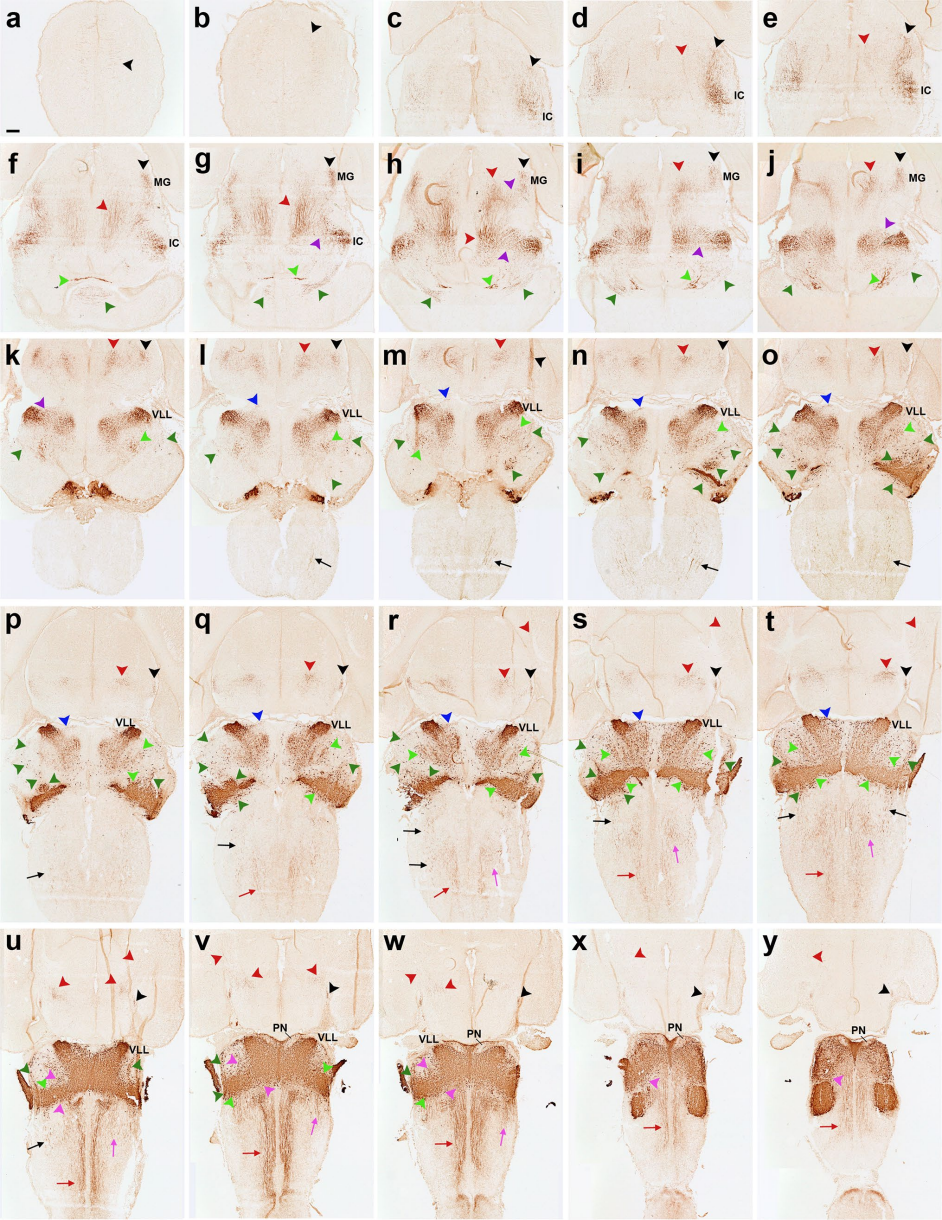
**a–y** Horizontal sections of an E16.5 *b1r4-Cre*/*YFP* brain, ordered from dorsal to ventral, and immunostained with anti-GFP antibody, show the lateral lemniscus (*black arrowheads*), the medial tegmental tract and its fibres entering the medial forebrain bundle (*red arrowheads*). A trigeminothalamic tract occupies a position intermediate between the lateral lemniscus and the medial tegmental tract in the *upper* brainstem (**g**–**k**, *violet arrowheads*). A superficial ascending tract (**l**–**t**, *blue arrowheads*), formed by a small ascending subpial axon bundle, seems to arise from a superficial group of dispersed labelled neurons localized just rostral to the pons. The trigeminal cerebellopetal tract (**f**–**w**, *light green arrowheads*) is formed by thick fibres that stream out from the trigeminal sensory column and approach the roof of the cerebellar plate behind the isthmus, also penetrating the cerebellar commissure (**f**). The vestibular cerebellopetal tract (**f**–**w**, *dark green arrowheads*) is formed by thinner fibres, originated from the vestibular complex and crossing the *midline* of the cerebellar nodule (vermis; **f**). Medial spinopetal tract fibres (**q**–**y**, *red arrows*) exit from the medial basal part of r4, probably originated from reticular and vestibular neurons. These fibres descend medially through the medulla and then adopt a superficial position in the ventral column of the spinal cord. The labelled lateral vestibulospinal tract (**l**–**u**, *black arrows*) originates from the r4-derived vestibular neurons in r4 (lateral vestibular nucleus) and from neighbouring cells previously migrated from r4 into r3 (superior vestibular nucleus) and r5 (inferior vestibular nucleus); it descends to the spinal cord, moving into the lateral column. The lateral trigeminal oro-spinal tract (**r**–**w**, *pink arrows*) courses intercalated between the medial descending tract and the lateral vestibulospinal tract. Dispersed labelled reticular neurons are found rostral and caudal to r4 (**u**–**y**, *pink arrowheads*). For abbreviations see “list of abbreviations”. *Scale bar* 200 µm

**Fig. 6:**
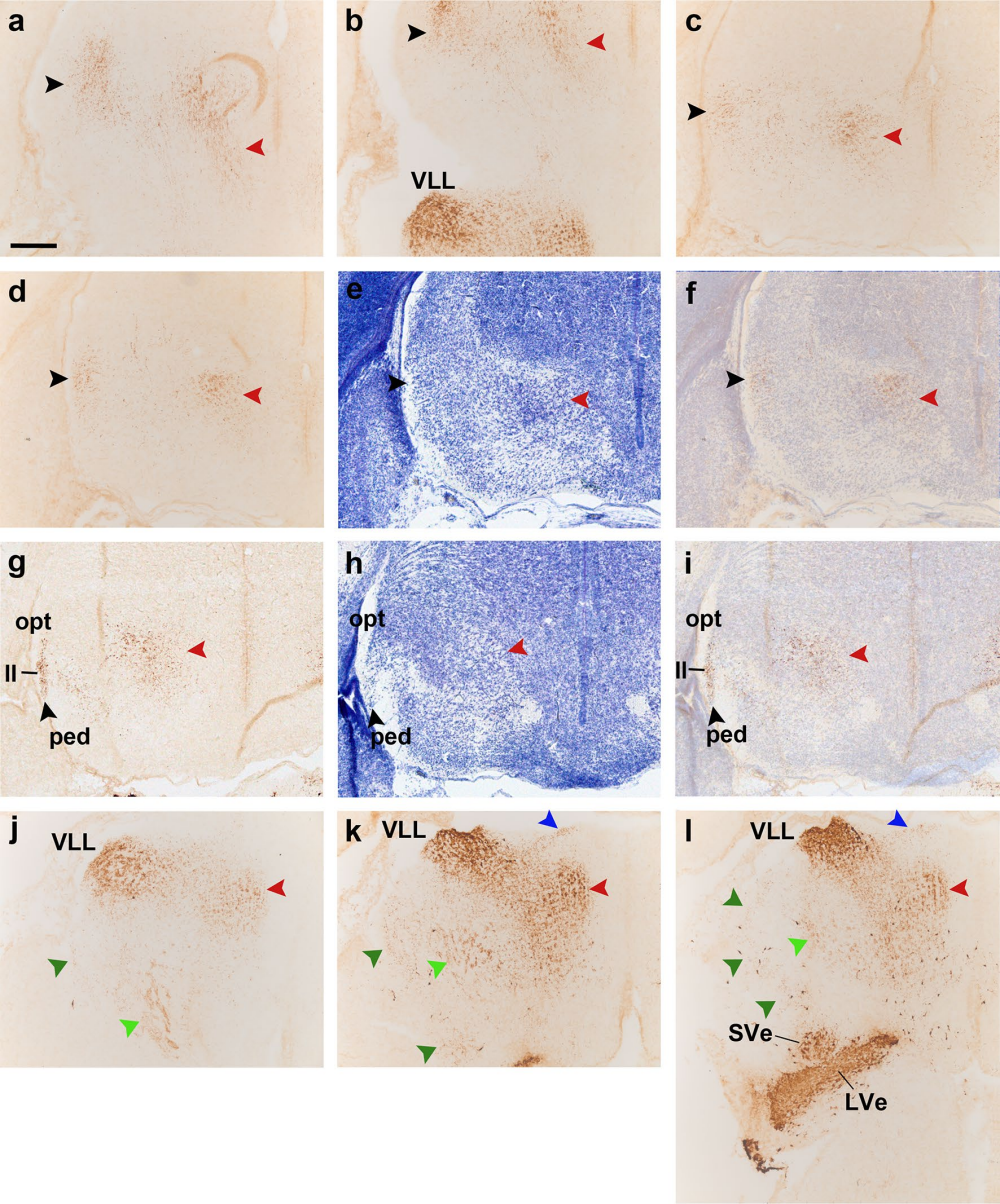
Details of horizontal sections of an E16.5 *b1r4-Cre*/*YFP* brain shown in Fig. 5, immunostained with anti-GFP antibody (**a–d, g, j–l**), two Nissl-stained sections adjacent to **d** and **g** (**e, h**) and the corresponding merged YFP/Nissl images (**f, i**). Due to the cephalic flexure, all these sections were cut roughly transversally to the diencephalon (in topological terms). The aim is to show labelled tracts coursing rostralwards through mesodiencephalic tegmental areas (**a–i**) and through the prepontine and pontine hindbrain tegmental region between the migrated VLL in r2 and the labelled r4 territory (**j–l**). (**a–c**) Midbrain territories cut dorsal, at level and rostroventral to VLL (VLL, **b**). The groups (**d–f**) and (**g–i**) each illustrate adjacent sections immunostained with anti-GFP antibody (**d, g**) and Nissl (**e, h**), jointly with the merged YFP/Nissl images (**f, i**). In the midbrain, the lateral lemniscus fibres are seen entering the inferior colliculus (**a**; *black arrowhead*), continuing into the brachium of the inferior colliculus (**b**; *black arrowhead*) and reaching the superficial primordium of the medial geniculate body in the thalamus (**c, d–f**; *black arrowheads*). Afterwards they adopt a subpial position just ventral to the optic tract and dorsal to the peduncular bundle (opt, ll, ped; **g–i**; *black arrowheads*) and extend in this position, adjacent to the alar–basal boundary, all the way to the supraoptic decussation (not shown). The ascending medial tegmental fibres (*red arrowheads*) stretch longitudinally through the midbrain (**a–c**) and diencephalic tegmentum (**d–i**) and also course thereafter within the basal hypothalamus (not shown). This tract is also observed coursing through the hindbrain basal plate (**j–l**; sections ordered from dorsal to ventral). The trigeminal cerebellopetal tract is formed by thick fibres (**j–l**, *light green arrowheads*). The vestibular cerebellopetal tract (**j–l**, *dark green arrowheads*) is formed instead by thin fibres originated from the vestibular area. The unidentified thin fibres composing what we call the ‘superficial tract’ is a separate small subpial ascending bundle marked with *blue arrowheads* (**k, l**). R4-derived vestibular neurons are observed within r3, forming a labelled patch within the superior vestibular nucleus (**l**). The migrated ventral nucleus of the lateral lemniscus also appears labelled (**b, j–l**). For abbreviations see “list of abbreviations”. *Scale bar* 200 µm

**Fig. 7:**
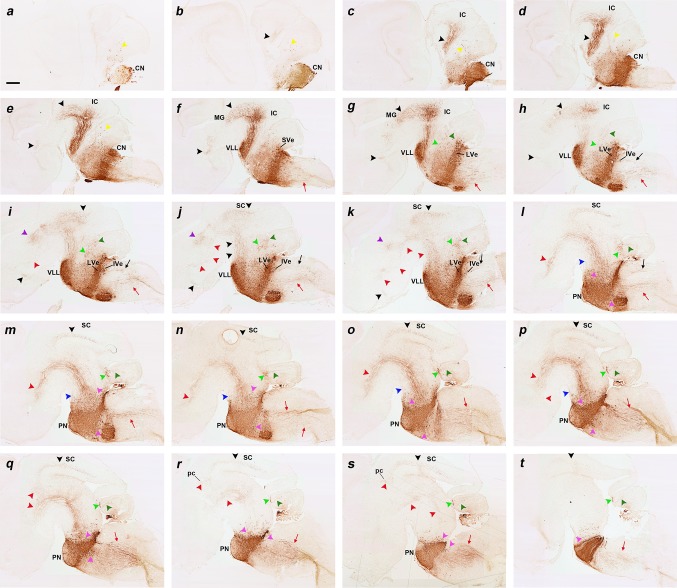
**a–t** Sagittal sections of an E18.5 *b1r4-Cre*/*YFP* brain, ordered from lateral to medial (ending at the *midline*) and immunostained with anti-GFP antibody. The YFP distinctly labels the r4 territory and r4 derivatives and related tracts. Most laterally, the labelled subdomain of the cochlear nuclei is shown (CN; **a–e**). The lateral lemniscus (*black arrowheads*) appears shortly afterwards (**b–e**) and shows profuse terminal innervation of the inferior colliculus (**e–h**), as well as of the deep stratum of the superior colliculus (**i–t**) and the brachium of the inferior colliculus extending into the diencephalon (**f–j**). In the thalamus, the lemniscal fibres course through the medial geniculate nucleus (MG; **f, g**) and approach the brain surface ventral to the optic tract, where they extend into the supraoptic decussation (not shown). The medial tegmental tract crosses the tegmentum of the prepontine hindbrain, midbrain, diencephalon and basal hypothalamus (**i–s;** *red arrowheads*), and contributes to the ascending medial forebrain bundle that reaches the telencephalon (not shown). Collaterals of this tract grow into the posterior commissure and neighbouring areas of the pretectum (*red arrowheads* next to pc; **q–s**). The trigeminothalamic tract apparently originates in the oral subnucleus of trigeminal descending column and extends into the posterior thalamic nucleus and the medial part of the ventrobasal complex of thalamus (**i–k**, *violet arrowheads*). The superficial ascending tract is a small labelled axon bundle that ascends subpially (**l–p**, *blue arrowheads*). The trigeminal cerebellopetal tract (*light green arrowheads*) and the vestibular cerebellopetal tract (*dark green arrowheads*) both reach the cerebellum. Fibres descending from r4 into the spinal cord form the medial spinopetal and lateral vestibulospinal tracts (*red* and *black arrows*, respectively). The lateral vestibular tract originates from r4-derived vestibular neurons (SVe, LVe and IVe in **f–k**). R4-derived reticular neurons are present in r3 and r5 (*pink arrowheads*). R4-derived oligodendrocytes invade the cerebellum (**a**-**e**, *yellow arrowheads*). For abbreviations see “list of abbreviations”. *Scale bar* 400 µm

**Fig. 8:**
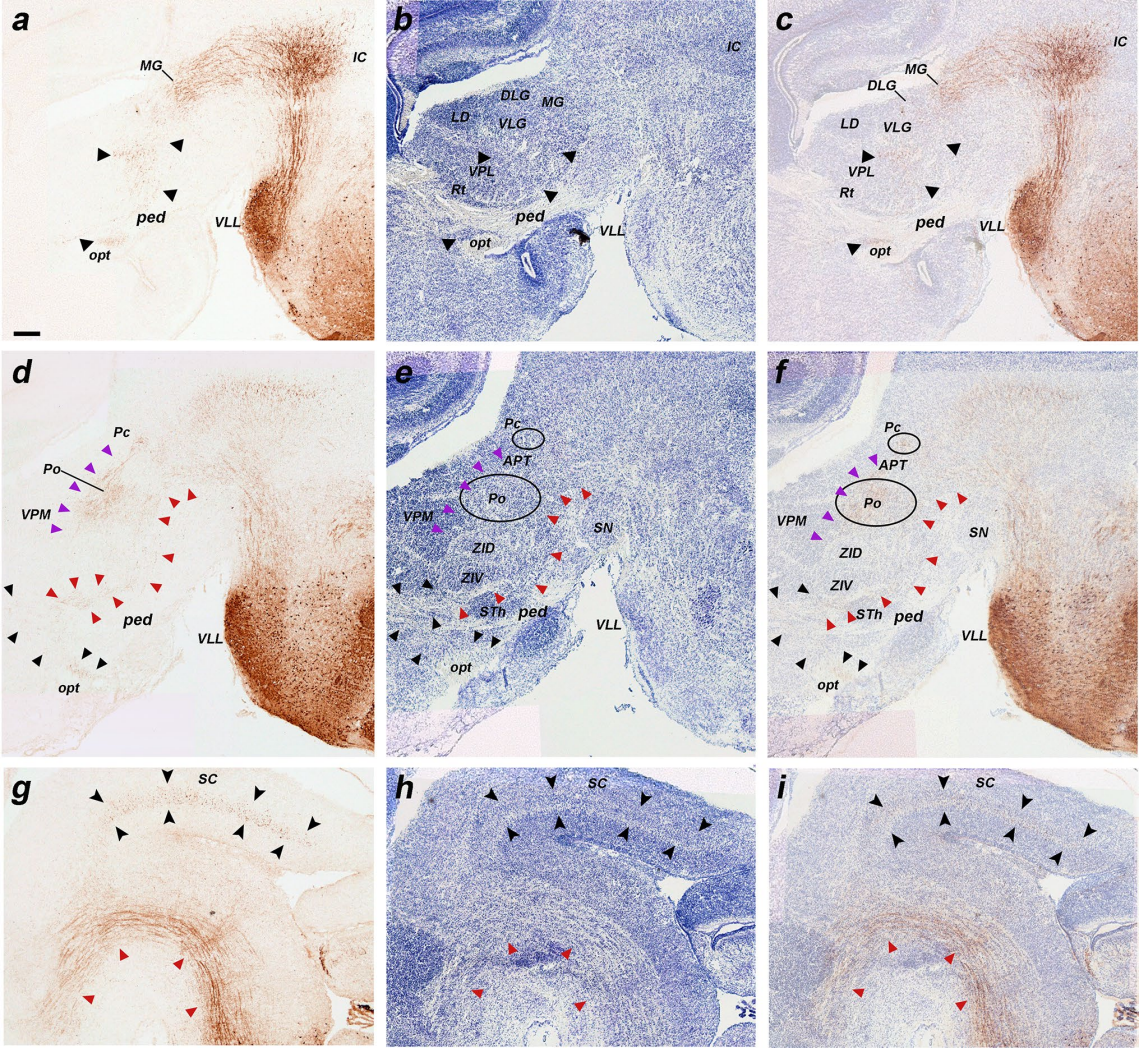
**a–i** Details at higher magnification of three of the sagittal sections of an E18.5 *b1r4-Cre*/*YFP* brain shown in Fig. 7, ordered from lateral to medial, and immunostained with anti-GFP (**a, d, g**), compared to Nissl-stained adjacent sections (**b, e, h**), and to merged YFP/Nissl images (**c, f, i**). The lateral lemniscus fibres penetrate profusely the inferior colliculus, entering radially its deep central part (**a–c**). A number of lemniscal fibres clearly extend through the brachium of the inferior colliculus (across midbrain and pretectum) to the thalamic medial geniculate nucleus (MG); more rostrally, the tract extends all the way to the supraoptic decussation (**a–c**, *black arrowheads*). These labelled lemniscal fibres course at the interface between the cerebral peduncle and the optic tract (opt, ped; rostralmost *black arrowheads* in **a–c**). Labelled fibres also penetrate the deep stratum of the superior colliculus (superficial to the periaqueductal grey) (SC; **g–i**, *black arrowheads*). The trigeminothalamic tract projects fibres to the posterior thalamic nucleus and the medial part of the ventrobasal complex (**d–f**, *violet arrowheads*). The medial tegmental tract crosses the tegmentum of the prepontine hindbrain, midbrain and diencephalon, reaching the hypothalamus (**d–f, g–i**; *red arrowheads*). For abbreviations see “list of abbreviations”. *Scale bar* 200 µm

**Fig. 9:**
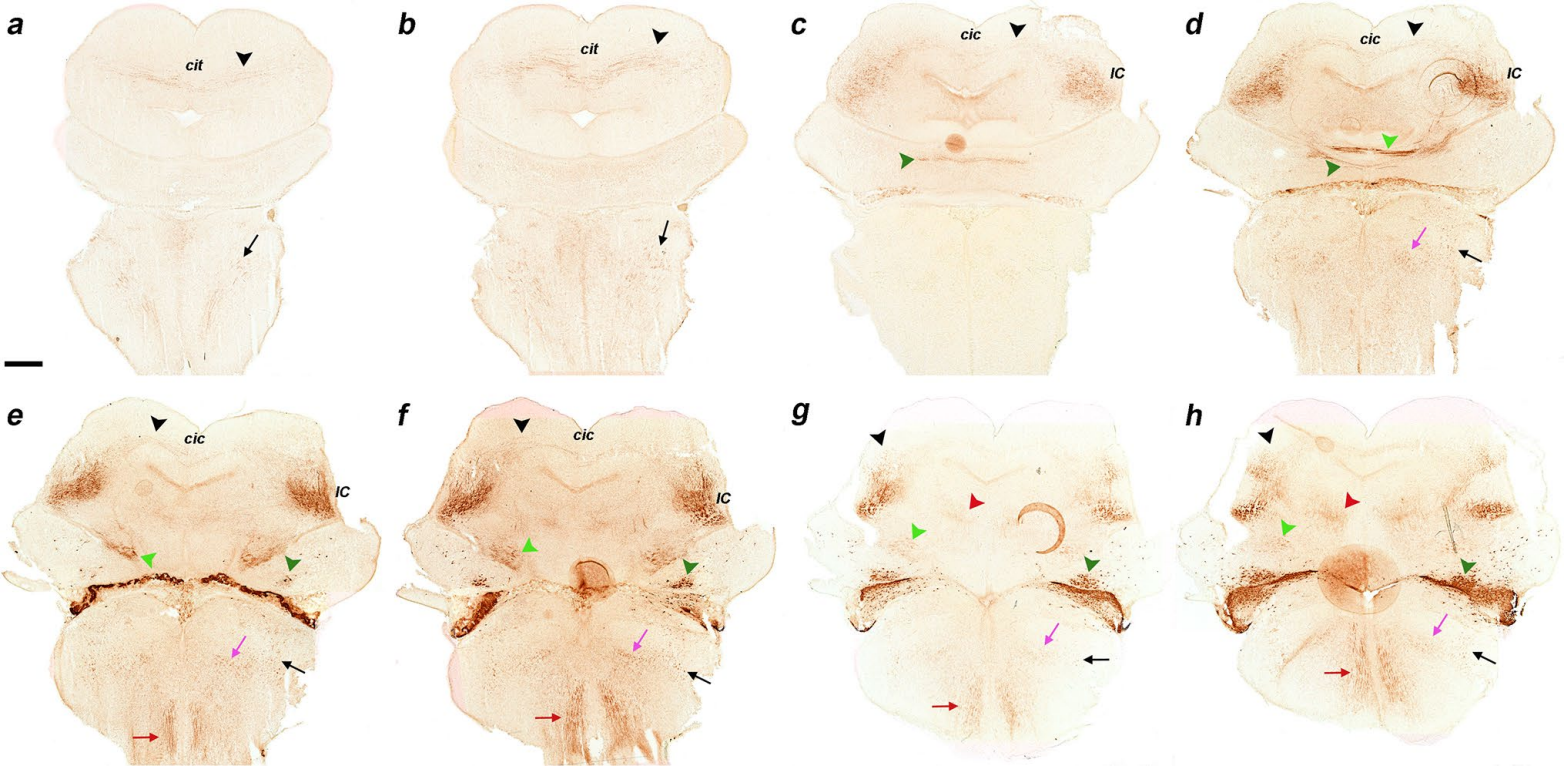
**a–h** Immunostaining with anti-GFP antibody on a series of horizontal sections through an E18.5 *b1r4-Cre*/*YFP* brain, ordered from dorsal to ventral. The YFP labels distinctly r4 and the r4 derivatives and related tracts. The lateral lemniscus (*black arrowheads*) reaches the deep part of the inferior colliculus (**e, f**) and extends fibres into the intercollicular commissure (**c, d**), the deep stratum of the superior colliculus (**b, c**) and the tectal commissure (**a, b**). The trigeminal cerebellopetal tract (*light green arrowheads*) reaches the cerebellum (**e–h**) and enters the cerebellar commissure (**d**). The vestibular cerebellopetal tract (*dark green arrowheads*) crosses the *midline* of the cerebellar nodule (**c, d**). Labelled fibres apparently derived from reticular and vestibular r4 neurons descend medially into the spinal cord forming the medial spinopetal tract (*red arrows*; **e–h**). The lateral vestibulospinal tract (*black arrows*) originates from r4-derived vestibular neurons (*dark green arrowheads*; **e–h**). The lateral trigeminal oro-spinal tract courses in an intermediate (lateral basal) tegmental position, intercalated between the medial spinopetal tract and the lateral vestibulospinal tract (*pink arrows*; **d–h**). For abbreviations see “list of abbreviations”. *Scale bar* 400 µm

**Fig. 10:**
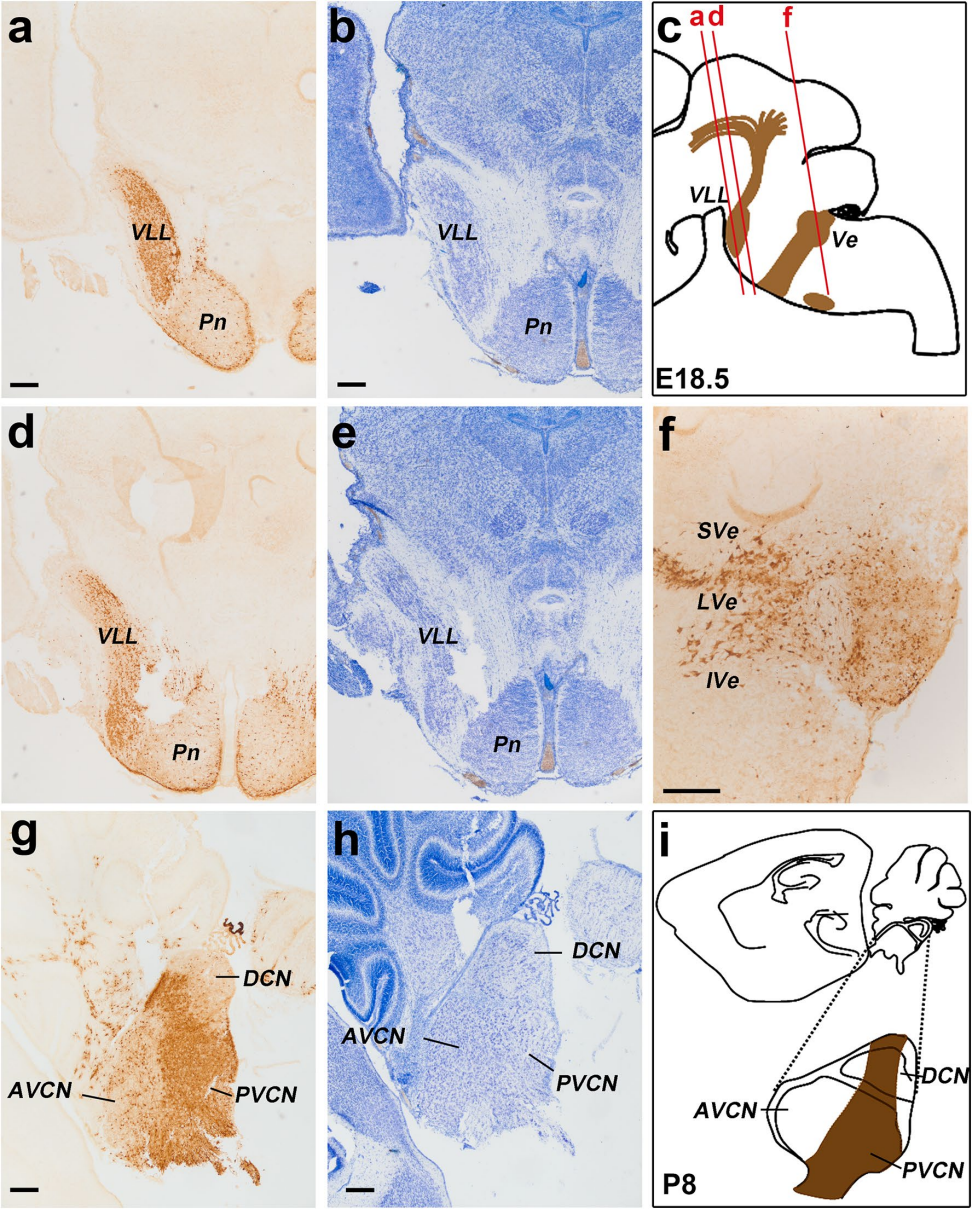
Detail of r4-derived parts of lemniscal, cochlear and vestibular nuclei in coronal sections at E18.5 (**a–f**), and of the cochlear complex labelling obtained in sagittal sections at P8 (**g, h**); a schematic view of the latter result is shown in (**i**). The *red lines* on the sagittal schema in **c** indicate the levels of coronal sections in **a, d** and **f**. We first show two pairs of adjacent coronal sections of an E18.5 *b1r4-Cre*/*YFP* brain stained with anti-GFP antibody (**a, d**) or Nissl (**b, e**), illustrating the aspect of the r4-derived ventral nucleus of the lateral lemniscus (VLL), which extends into alar r2 beyond the rostral pontine formation in r3. In a more caudal coronal section (**f**), the r4-derived lateral vestibular nucleus (LVe) is continuous with migrated similar cells displaced into r3 and r5, corresponding to the SVe and IVe nuclei, respectively. (**g–i**) Adjacent sagittal sections of a P8 *b1r4-Cre*/*YFP* brain stained with anti-GFP (**g**) and Nissl (**h**) showing labelling in the cochlear nuclear complex. The sagittal section schema (**i**; compare also with Fig. 11) displays the position of the cochlear complex in the P8 brainstem and emphasizes at higher magnification its r4-derived components. R4 contributes to most of the posteroventral cochlear nucleus (PVCN) and to the rostroventral part of the dorsal cochlear nucleus (DCN), leaving the anteroventral nucleus (AVCN) and the polar rostral and caudal parts of the dorsal nucleus (DCN) unlabelled. For abbreviations see “list of abbreviations”. *Scale bar* 200 µm

**Fig. 11:**
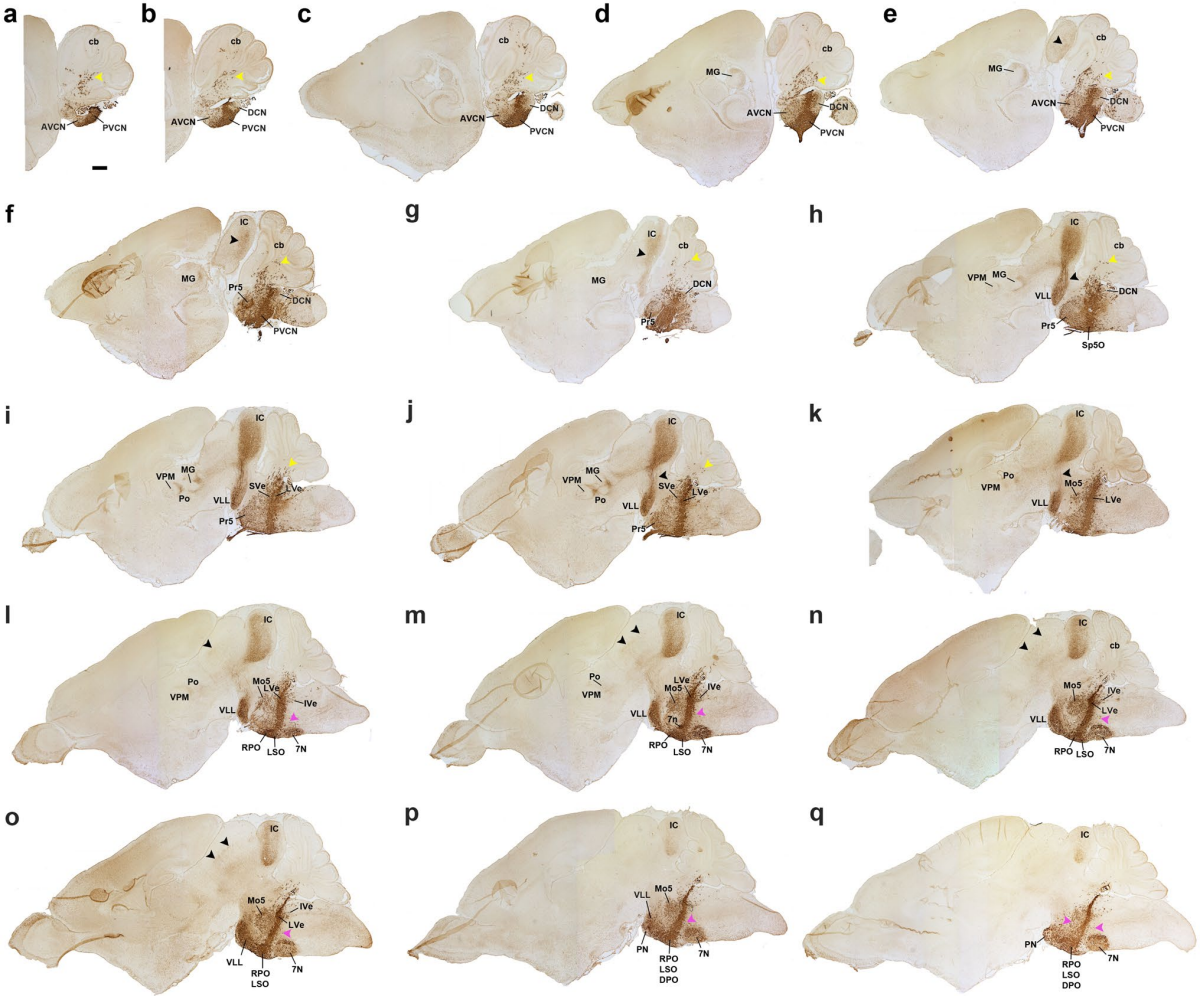
**a–q** Immunostaining with GFP antibody of a series of sagittal sections of a P8 *b1r4-Cre*/*YFP* brain, ordered from lateral to medial. The YFP labels massively the r4 territory and the r4 derivatives spreading outside of r4. YFP-positive glial cells have migrated into the cerebellum (**a–j**, *yellow arrowheads*). Most of the posteroventral cochlear nucleus and the rostroventral part of the dorsal cochlear nucleus originate from r4 (PVCN; DCN; **a–h**). The r4-derived lateral vestibular nucleus (LVe) lies strictly within r4, but some of its cells are displaced into the neighbouring SVe and IVe nuclei in r3 and r5 (**i–o**). Dispersed labelled reticular neurons are located in r2, r3 and r5 (**l–q**, *pink arrowheads*). R4-derived postmigratory lemniscal neurons form the ventral nucleus of the lateral lemniscus (which projects fibres to the inferior colliculus and elsewhere) (**e–q**, *black arrowheads*). The labelled lemniscal tract is formed initially by crossed fibres of r4 projection neurons in the contralateral cochlear nuclei and incorporates also ipsilateral r4-derived VLL fibres. Many lemniscal fibres apparently extend collateral branches through the brachium of the inferior colliculus to the medial geniculate nucleus (**d–j**) and beyond that into the supraoptic decussation (not shown). YFP-positive lateral lemniscal fibres also reach the deep stratum of the superior colliculus (**l–o**). The trigeminothalamic tract is formed by fibres that reach the posterior thalamic nucleus and the ventral posteromedial nucleus (**h–m**). Rostral to the r4-derived facial nucleus, part of the lateral superior olive and the dorsal periolivary area are labelled, as is, more anteriorly, the rostral periolivary region (LSO, DPO, RPO; **l**–**q**). YFP-positive oligodendrocytes populate the pontine nuclei (pontine *grey* and reticulotegmental nuclei of pons) (**p, q**) as well as the area of the trigeminal motor nucleus (**k**–**p**). For abbreviations see “list of abbreviations”. *Scale bar* 400 µm

**Fig. 12:**
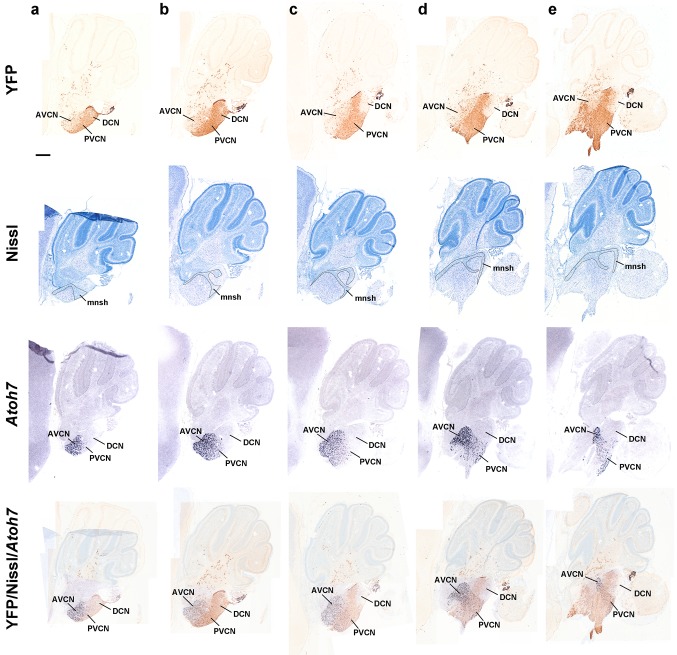
Details at P8 of the r4-derived part of the cochlear complex and oligodendrocytes migrated to the cerebellum. **a**–**e** *Vertical panels* each composed of 4 images corresponding to anti-GFP, Nissl, in situ *Atoh7* and the corresponding merged YFP/Nissl/*Atoh7* images on a series of adjacent lateral sagittal sections of a P8 *b1r4-Cre*/*YFP* brain. R4 distinctly contributes to the rostroventral part of the dorsal cochlear nucleus and the major part of the posteroventral cochlear nucleus, while only scattered YFP^+^ cells are distributed in the anteroventral cochlear nucleus, which is strongly and differentially positive for *Atoh7*. R4 does not contribute to the granule cells of the microneuronal shell (*outlined* in the Nissl images). Note also YFP^+^ oligodendrocytes migrated from r4 into the white matter of the cerebellum. For abbreviations see “list of abbreviations”. *Scale bar* 400 µm

**Fig. 13:**
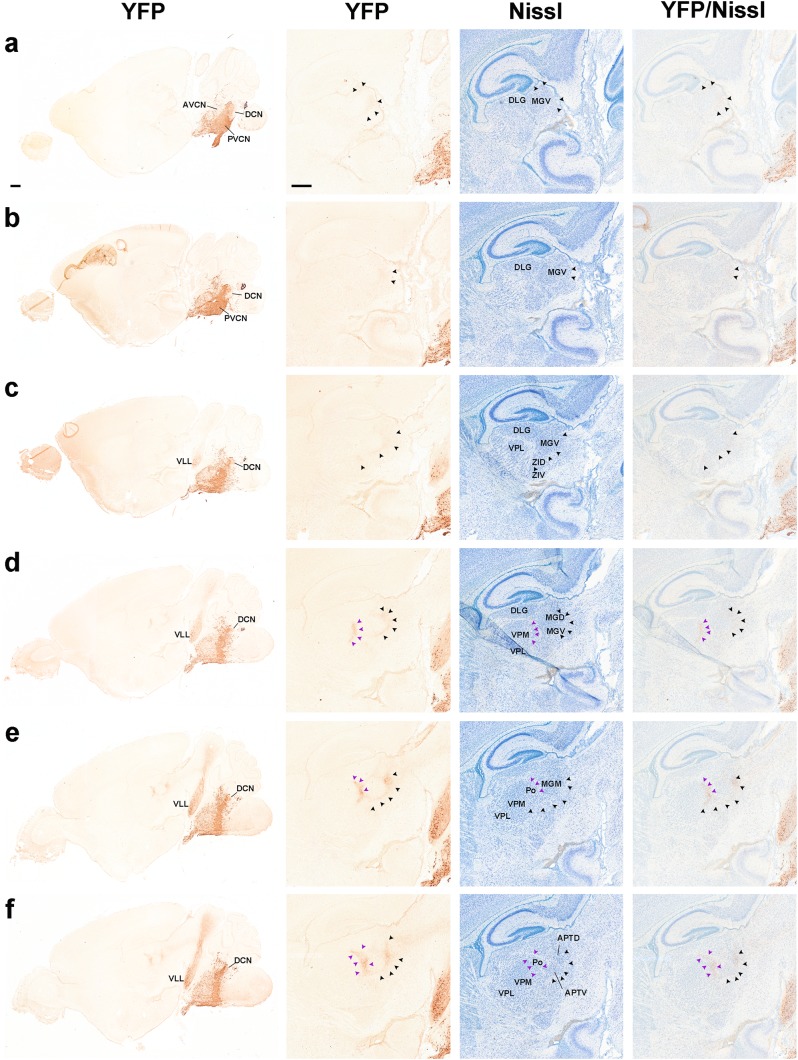
Illustration of r4-derived fibres projecting to the thalamus at P8. **a**–**f** Immunostaining with anti-GFP antibody on series of adjacent sagittal sections of a P8 *b1r4-Cre*/*YFP* brain, ordered from lateral to medial (*left* panels marked YFP in each set of four). High magnification of the thalamic region is shown in the next three images at each level, from adjacent sections stained with anti-GFP and Nissl and the corresponding merged YFP/Nissl images (*right side* images) to locate the r4-derived fibres projecting into the thalamus. The lateral lemniscal fibres (*black arrowheads*) course longitudinally roughly through the area occupied by the prospective medial, ventral and dorsal subdivisions of the medial geniculate nucleus in the thalamus. R4-derived trigeminothalamic fibres (*violet arrowheads*) project to the posterior thalamic nucleus and the medial part of the ventrobasal complex. For abbreviations see “list of abbreviations”. *Scale bar* 400 µm

**Fig. 14:**
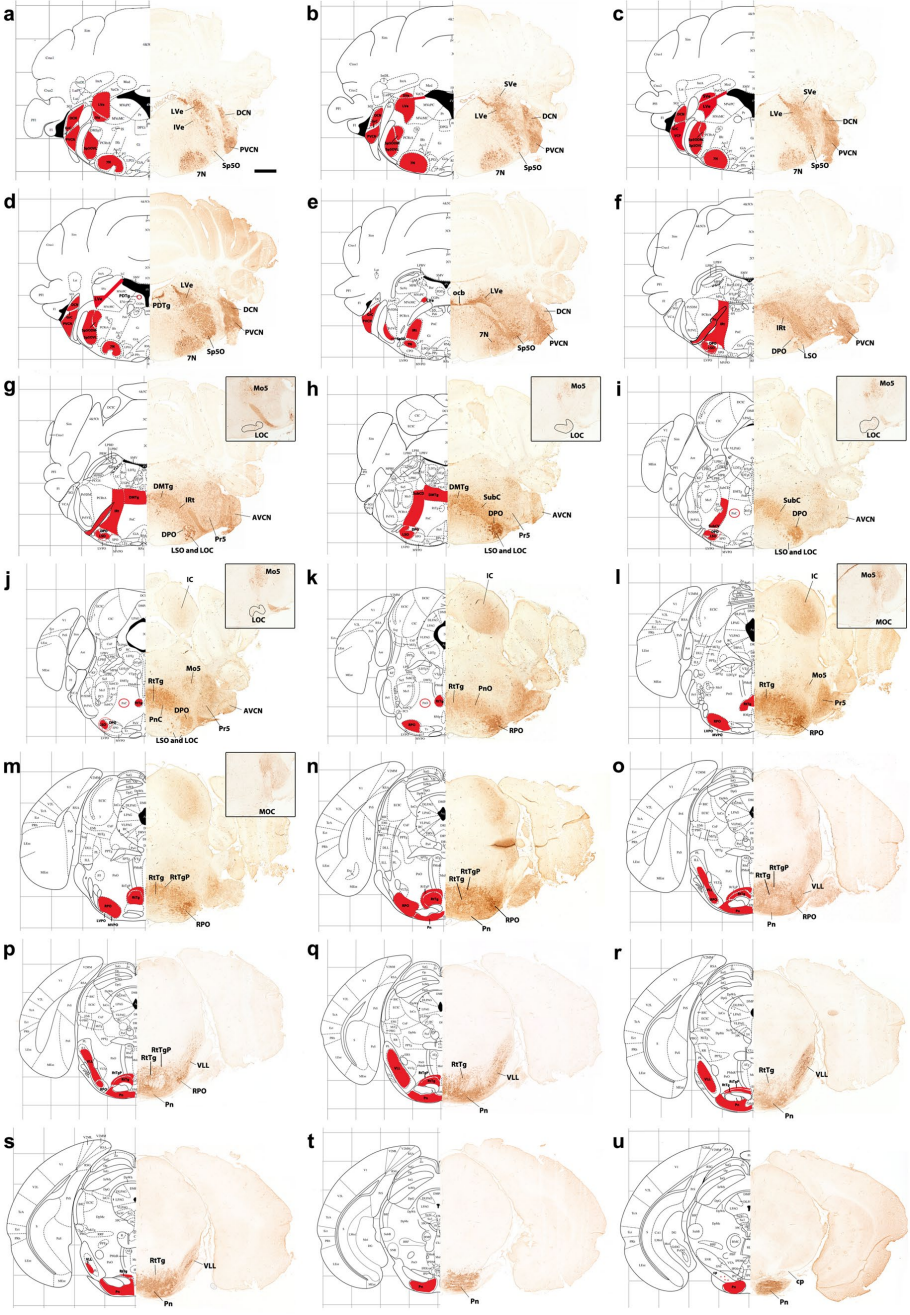
Adult results. **a**–**u** Hemisections of coronal sections of *b1r4-Cre*/*YFP* adult mice immunostained with anti-GFP antibody, ordered from caudal to rostral, and juxtaposed to schematic coronal sections at similar levels from the Mouse Brain Atlas (Paxinos and Franklin 2001). The r4-derived domain and all r4 derivatives are YFP positive and are mapped in *red* on the corresponding schematic sections. YFP-positive r4 derivatives include the following: the facial motor nucleus (**a**-**e**); the lateral vestibular nucleus and neighbouring IVe and SVe nucleus (**a**-**e**); the oralis part of the spinal trigeminal nucleus (**a**–**e**); most of the posteroventral cochlear nucleus and the rostroventral part of the dorsal cochlear nucleus (**a**–**f**); the ventral nucleus of lateral lemniscus (VLL; **o**–**s**); the lateral superior olive (LSO; **f**–**j**); the dorsal periolivary area (DPO) (**f**–**j**); the rostral periolivary region (RPO; **k**–**p**); choline acetyltransferase (ChAT)-positive lateral (*inset* in **g**–**j**; LOC) and medial (*inset* in **l, m**; MOC) olivocochlear neurons; the intermediate reticular nucleus (IRt; **e**–**g**); the posterodorsal tegmental area (PDTg; **c, d**); a part of nucleus subcoeruleus (SubC) (**h, i**); caudal (**i, j**) and oral (**k**) parts of the pontine reticular nucleus; oligodendrocytes in the reticulotegmental (**j**–**s**) and pontine grey nuclei of the pons (**n**–**u**) and YFP-positive fibres in the cerebral peduncle (**u**). For abbreviations see “list of abbreviations”. *Scale bar* 800 µm

**Fig. 15:**
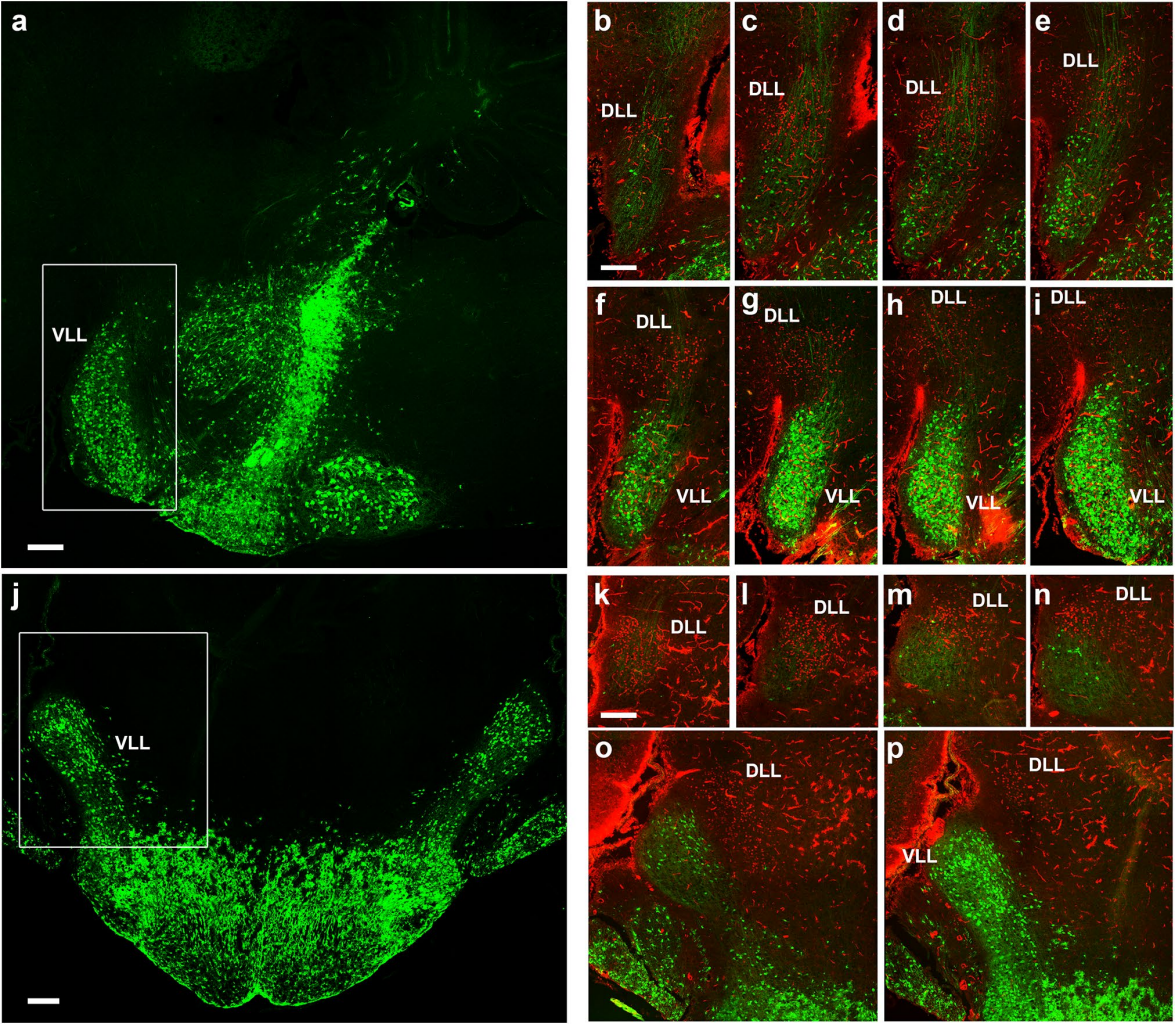
Fluorescent YFP observations on the ventral nucleus of the lateral lemniscus in the P8 *b1r4-Cre*/*YFP* brains. Panoramic sagittal and horizontal sections through r4 are shown in (**a**) and (**j**). The section in (**a**) illustrates the densely labelled r4 domain (including in its midst the dense packets of efferent facial nerve fibres), as well as the caudally migrated facial motoneurons in r6 (note their large size) and the r4-centred contribution to pontine grey, reticular formation, trigeminal, vestibular and cochlear columns, accompanied by cells spreading variously rostral and caudal to r4 (note oligodendrocytes in the area of the trigeminal motor nucleus (r2 and r3), but no motoneurons analogous in size to the facial ones); finally, the migrated VLL extending rostral to r4 is clearly visualized, stretched along the course of the lateral lemniscus. **b**–**i** Series of adjacent lateral sagittal sections from the same brain, ordered latero-medially, double-labelled for cytoplasmic and axonal YFP (*green*) and *Pax7* immunoreaction in cell nuclei (*red*; note blood vessels also react to *Pax7*). *Pax7* reaction characterizes the DLL nucleus originated in alar r1, whereas YFP characterizes the basal r4-derived VLL nucleus in r2–r3 (compare **a**). The series illustrates their mutual topography. In general, *green*-labelled VLL neurons do not overlap with *red*-labelled DLL neurons, though *green* fibres pass through DLL. There is a vaguely delimited space between DLL and the massive VLL, where only few *green*-labelled neurons are found; this probably corresponds to the largely unlabelled ILL nucleus (**b**–**f**). (**j**) The horizontal section passes through the r4-derived pontine region (presumably mostly glial cells; note that basilar pontine neurons remain unlabelled, being derived jointly with the reticulotegmental nucleus from rhombic lip areas caudal to r4), and the rostrally extending VLL population in r2–r4. **k**–**p** Dorsoventral series of horizontal sections of the same brain, double-labelled with YFP (*green*) and anti-Pax7 antibody (*red*), illustrating in this plane the relative rostral location of the Pax7-positive DLL (*red*; **k**–**n**) with respect to the poorly YFP-labelled ILL area (diffuse *green* fibres, few *green* cells; **l**–**o**), and the strongly *green* fluorescent VLL (**p**). *Scale bar* 400 µm

**Fig. 16:**
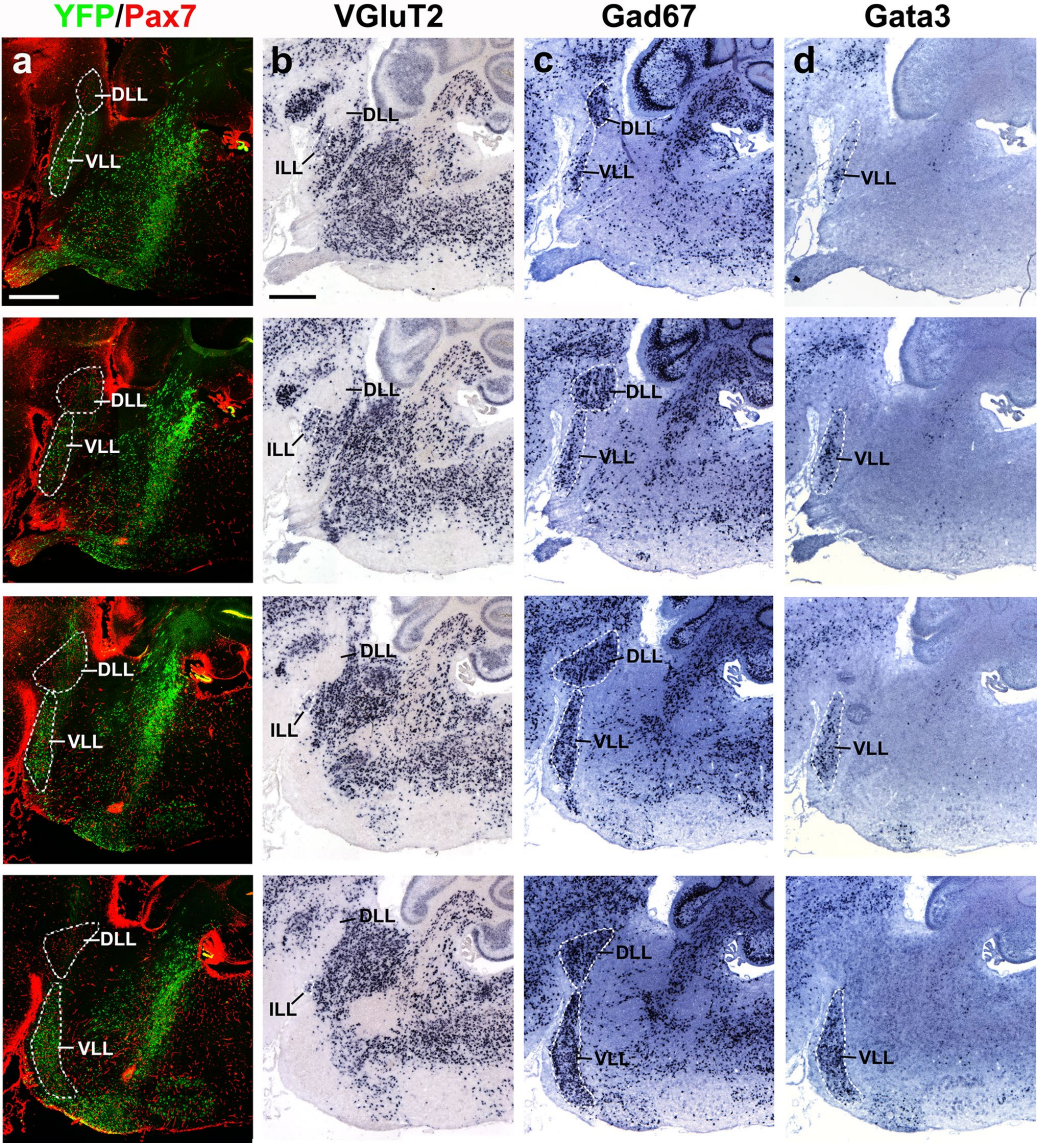
Series of adjacent lateral sagittal sections of a P8 b1r4-Cre/YFP brain stained with YFP/*Pax7* (**a**), *VGluT2* (**b**), *Gad67* (**c**) and *Gata3* (**d**). A *dashed white line* delimits the ventral (VLL), intermediate (ILL) and dorsal (DLL) nuclei of the lateral lemniscus. The DLL lies in r1 and is labelled by *Pax7* and *Gad67*, being negative for *VGluT2* and *Gata3*; this suggests a GABAergic/glycinergic phenotype of DLL. Separately, in the absence of *Pax7* signal, YFP, *Gad67* and *Gata3* label the r4-derived VLL neurons in r2 and r3, which accordingly also have a GABAergic/glycinergic phenotype. *Green* fluorescent-labelled VLL neurons do not overlap with *red*-labelled DLL neurons, though *green* fibres pass through DLL. There is a vaguely delimited space between DLL and the massive VLL, where scarce YFP^+^/*Gad67* ^+^/*Gata3* ^+^/ neurons are found; in this zone, a group of *VGluT2* ^+^ neurons form the ILL nucleus, which would accordingly consist of glutamatergic neurons. *Scale bar* 400 µm

Another sizeable, though less compact, exiting stream of labelled r4 cells emerges rostrally from the basal longitudinal zone after E10 (Fig. 2d–f). These cells are preceded by a growing ascending tract that was identified at older stages as the lateral lemniscus (Fig. 1b/bʹ–d/dʹ, h/hʹ–k/kʹ, 2b–g; this tract carries crossed fibres from the cochlear nuclei, which comprise an r4 module, plus uncrossed fibres of the largely r5-derived superior olivary complex and of the interstitial cell populations known as bed nuclei of the lateral lemniscus; Malmierca and Merchán 2004). The migrating cells move along or inside this superficial tract, which first courses obliquely rostralwards through the basal plate of r3 and then passes into the alar plate to ascend through the prepontine alar hindbrain (r2, r1, isthmus) into the inferior colliculus of the midbrain. By E12.5, the cells have advanced about 140 micrometres beyond the rostral boundary of r4, apparently lying still mainly within r3 (Fig. 2e, f). By E14.5, the pioneering elements of this cell population extend farther along the growing lateral lemniscus, stopping roughly at the estimated boundary between r2 and r1 (Fig. 3b–g). At subsequent stages (we examined E16.5, Figs. 4, 5, 6; E18.5, Figs. 7, 8, 9, 10; P8, Figs. 10, 11, 12, 13; adult Fig. 14), the migrated labelled cell population lying interstitial to the lateral lemniscus remains stretched between r2 and r4 and clearly correlates topographically with the ventral nucleus of the lateral lemniscus (VLL; Di Bonito et al. 2013a; Ito et al. 2011; Malmierca and Merchán 2004; Figs. 4b–f, 7f–k, 11h–p, 14o–s). No labelled cells were found at the more rostral r1 locus of the *Pax7*- and *Gad67*-positive dorsal nucleus of the lateral lemniscus (Figs. 15b–i, k–p, 16; Moreno-Bravo et al. 2014; Allen Developing Mouse Brain Atlas). The green-labelled cells *Gad67*- and *Gata3*-positive clearly represent the majority of neurons in the VLL nucleus, with few green neurons spread in the space between DLL and the massive VLL occupied by the *VGluT2*-positive intermediate lemniscal nucleus (ILL) (Figs. 10a, b, d, e, 14o–s, 15, 16).

#### Vestibular complex

At dorsoventral levels corresponding to the vestibular column, a number of labelled neurons exit r4 rostrally and caudally; they come to a stable new position in r3 and r5, respectively, always within the vestibular complex. The r4 vestibular elements invading r5 (i.e. the inferior vestibular nucleus) first appear at E10.5 (Fig. 1a/aʹ, e/eʹ), and their number increases at E11.5 (Fig. 1g/gʹ, k/kʹ, l/lʹ) and E12.5 (Fig. 2g–i). The migrated cell group is constituted by distinct large stellate neurons which remain visible at E14.5 (Fig. 3d, e, k), E16.5 (Figs. 4c, 5n–t), E18.5 (Figs. 7h–k, 9g, h, 10 f), P8 (Fig. 11l–o) and the adult (Fig. 14a). We found no labelled migrated cells within the inferior vestibular nucleus at r6 levels, or more caudally. On the other hand, the r4 vestibular elements invading the r3 vestibular column module (i.e., the superior vestibular nucleus) start to sort out of r4 between E10.5 and E12.5 (Fig. 1a, e/eʹ, l/lʹ, 2h, i). They incipiently aggregate within r3 at E14.5 (Fig. 3a, b) and later form a dense, well-circumscribed and large-celled aggregate at E16.5 (Figs. 4a, 5n–t, 6l), E18.5 (Figs. 7f, 9f–h, 10f), P8 (Fig. 11i, j) and the adult (Fig. 14b, c). We observed some sparser labelled cells more rostrally in the vestibular column (superior vestibular nucleus), which possibly lie within r2 (Fig. 5m).

#### Cochlear complex

At E18.5 and postnatally, we observed some dispersed labelled neurons in the ventral and dorsal cochlear nuclei outside of the central labelled wedge representing the r4 cochlear column module (Figs. 9g, h, 10g, 11a–h, 12a–e). The apparently tangentially migrated population is more important in the anteroventral cochlear nucleus (rostral to r4). Notably, r4 does not contribute to the granule cells of the microneuronal shell of the cochlear complex (Fig. 12a–e; see also Di Bonito et al. 2013a; their Fig. 1J).

#### Reticular formation

After E10.5, there appear some dispersed labelled reticular neurons in r5, usually close to r4 (Fig. 1i/iʹ–k/kʹ; 2b–h; 3c–i; 4d–i; 5u–y; 7l–s; 11l–q). Dispersed labelled reticular elements were also observed after E14.5 rostral to r4, mainly medial to the trigeminal sensory column in r3 and r2, or in a neighbouring paramedian tegmental position (Figs. 3d–i, 4d–i, 5u–w, 7l–t, 11l–q).

#### Superior olivary complex

Labelled neurons were observed in r5, within part of the S-shaped lateral superior olive, at P8 (see Di Bonito et al. 2013a; their Fig. 1F and S7) as well as in the adult (Fig. 14f–j).

The lateral olivocochlear (LOC) and medial olivocochlear (MOC) efferent neurons (Brown and Levine 2008; Simmons 2002) are part of the inner ear efferent system (IEE); they are born in ventral (basal) r4 and are identified as migrated into the r5 territory by their characteristic *ChAT* expression (Di Bonito et al. 2013a). They lie in or near the lateral superior olive (LSO; Fig. 14g–j and inset), or in the rostral and ventral periolivary region (Fig. 14l, m and inset).

#### Cerebellum

The cerebellum is a derivative of the isthmus (vermis) and r1 (hemisphere). We observed a number of small labelled cells in the cerebellar white matter, which we identified as oligodendrocytes (Buffo and Rossi 2013) migrated out of r4 at E18.5 (Fig. 7a–e), P8 (Figs. 11a–j, 12a–e; see also the merged YFP/Nissl images) and in the adult (Fig. 14a–k).

#### Periventricular tegmentum

R4 apparently contributes via rostralward tangential migration to the dorsomedial tegmental nucleus (DMTg), which is found postnatally within caudal r1 or r2 (Fig. 14g, h). Labelled neurons also characterize the nucleus subcoeruleus, found caudally to the locus coeruleus, which is restricted to r1 (SubC; Fig. 14h, i).

### Tangentially migrated medullary derivatives invading r4 mix with local oligodendroglia

The medullo-pontine migration of r6–r8 rhombic lip precerebellar cells that eventually builds up the basilar pontine nuclei and the reticulotegmental nucleus in r3 and r4 is very well known and will thus not be described in detail here. The relationship of r4 with the prospective pons only begins to be appreciated after E16.5, when the unlabelled pontine migration stream reaches its target locus (Figs. 4c–f, 5v–y). Although the migrated neuronal population of the basilar pons primordium is largely unlabelled, it contains internally after E16.5 and postnatally a thinly dispersed labelled population as well as an outer crust of labelled cells, which may be glial in nature (Figs. 4d–i, 5v–y, 7l–s, 11p, q). This result was expected, since the basilar pontine neurons migrate in from more caudal parts of the rhombic lip (Altman and Bayer 1978) and thus should be unlabelled in our material, and a large production of oligodendrocytes occurs in the pontine r4 territory (Miguez et al. 2012). The scattered small (presumably glial) labelled pontine cells are present exclusively inside the r4 pontine area, being absent in the pontine sector belonging to r3 (compare Fig. 10a, b). The relatively small r3 pontine sector can be best identified at E18.5 and P8 (Figs. 7l–s, 10a, b, d, e, 11p, q). In the adult, YFP-labelled oligodendrocytes strongly populate the pontine grey (Pn) (Fig. 14n–u), as well as the central and pericentral parts of the reticulotegmental nucleus, which derives likewise from the bulbopontine migration (RtTg, RtTgP; Fig. 14j–s).

### Radially migrated r4 derivatives

At early stages, the identification of specific derivatives is handicapped both by their immaturity and the dense labelling obtained after the GFP immunoreaction. The facial and vestibulocochlear roots are useful r4 landmarks, since they are enclosed by the transverse boundaries of this segment. The r3/r4 boundary passes internally rostral to the facial knee and just caudal to the trigeminal motor nucleus (the trigeminal motor nucleus is placed in r2 and r3, as we corroborated in parallel ChAT-immunoreacted and Nissl-stained sectioned material; not shown). The r4/r5 limit passes rostral to the abducens nucleus lying in r5 (as we verified with ChAT-immunoreacted and Nissl-stained parallel series; not shown).

Our interpretation of subsequent stages, in which the mantle layer starts to reveal structures more or less distinguishable one from another, relies on the assumption of a well-known columnar structure within alar r4, which is shared with the neighbouring neuromeric parts of the hindbrain; that is, we assumed that structural sectors labelled within r4 that are serially continuous with characteristic sensory columns identified outside r4 represent r4 components or modules of these columns. We accordingly found, as expected, a selectively labelled r4 sector intercalated in the cochlear, vestibular and trigeminal columns, as well as in the reticular formation.

The pattern found at the cochlear column was clearest in lateral sagittal sections at P8 (Figs. 10g–i, 11a–h, 12), in which a wedge-shaped labelled sector intersects both the ventral and dorsal nuclei of the cochlear complex, consistently with adult coronal sections (Fig. 14a–j). Most of the posteroventral cochlear nucleus appears labelled (which agrees with the classic observation that the cochlear nerve root enters through it; Lorente de Nó 1981; Malmierca and Merchán 2004), whereas the anteroventral cochlear nucleus, which is differentially positive for *Atoh7*, is largely unlabelled, excepting the dispersed labelled cells mentioned above (Fig. 12). A rostroventral part of the dorsal cochlear nucleus also becomes densely labelled, while the caudodorsal part and the extreme rostroventral part remain unlabelled, except for few dispersed elements (Figs. 10g, 11a–h, 12). More medial sagittal sections and horizontal sections show a progressive lateromedial thinning of the labelled wedge intercalated within the cochlear complex as the latter extends medialwards periventricularly (Figs. 5m–o, 7n, 9g, h). Lateral sections show that the labelled wedge extends uninterruptedly dorsalwards into the rhombic lip and the attached labelled portion of the choroidal tela of the IV ventricle (Fig. 4a).

The findings for the vestibular column are already implicit in our description of the tangentially migrated components of this column. Rhombomere 4 apparently generates a good number of large multipolar vestibular neurons, which form locally the lateral vestibular nucleus, whereas the labelled multipolar vestibular cells that invade r3 and r5 are integrated within the superior vestibular and inferior vestibular parts of the same column (Figs. 10f, 11i–o, 14a–e).

The vestibular efferent neurons (VEN) born in basal r4 move into a position dorsal to the facial nerve genu within r4-derived territory (Martinez-de-la-Torre et al. 2017; Simmons 2002; see also Di Bonito et al. 2015; their Fig. 5a–c), and their axons enter the vestibulocochlear nerve together with those of the lateral and medial olivocochlear neurons.

The alar r4 domain labelled ventral to the vestibular column corresponds to the trigeminal column (Fig. 5p–w). This sector represents a segmental module of the oral subnucleus of the descending trigeminal nucleus (Sp5O) (Fig. 14a–e), which is divided into dorsomedial and ventrolateral parts (Sp5ODM, Sp5OVL). This interpretation rests on the assumption that the main trigeminal sensory nucleus (Pr5) is restricted to r2 and r3 correlatively with the ascending branch of the trigeminal nerve root (Oury et al. 2006). At r4 levels, no part of the viscerosensory column is present yet (it starts at r7 level; Martinez-de-la-Torre et al. 2017).

Since the branchiomotor elements of r4 migrate tangentially away into r6 (the facial nucleus), while the parasympathetic preganglionic superior salivatory nucleus supposedly originates and remains in r5, it can be assumed that the rest of labelled r4 tegmentum found ventral to the trigeminal column and dorsal to the basilar pons primordium should largely contain lateral, intermediate and medial populations of the r4 reticular formation. The rostral part of the intermediate reticular nucleus (IRt) lies in the YFP-positive region in adult coronal sections (Fig. 14e–g). R4 possibly also contributes to the caudal and oral pontine reticular nuclei of the medial reticular formation (PnC, PnO; Fig. 14i–k). We already commented above that at this level the boundaries of r4 are made fuzzy by the dispersion of some reticular neurons out of r4 into neighbouring areas of r3 and r5. Rostral to the facial nucleus, in a subpial position, the most lateral part of the lateral superior olive (LSO) (Fig. 14f–j) and the dorsal periolivary area (DPO) (Fig. 14f–j) are labelled, implying that they contain r4-derived cells; it is unclear whether these cells are located within r4 territory (being radially migrated), or have migrated tangentially into r5 (the SOC largely lies in r5). In a more rostral position, the rostral periolivary region (RPO) is strongly and entirely YFP positive, and therefore we ascribe it to r4 proper (Fig. 14k–p), jointly with the r4-located caudal portion of the VLL nucleus. Labelled LOC and MOC efferent neurons seem to be located both in r5- and r4-derived territory (Fig. 14g–j, l, m and insets; see also Di Bonito et al. 2013a; their Fig. 1f and S7).

The r4-derived radial histogenetic domain is delimited by the choroidal roof dorsally and the midline raphe ventrally.

The flattened neuroepithelial cells of the r4 choroidal roof also appear intensely labelled; they form a distinct transverse labelled band across the hindbrain roof (Figs. 1f, j–l, 2c–i, 3a–e, h, p, 4a–j, 7i–t, 9e, f, 11, 12).

The hindbrain floor plate apparently lacks neurogenetic activity (no known neuronal derivatives) and its neuroepithelial cells directly differentiate into radial glial cells. Their cell bodies lie in the local median ventricular zone, and they have basal (radial) cytoplasmic processes that reach the medioventral pial surface. Collectively, these cells build a median palisade that is conventionally known as “the hindbrain median raphe”. Labelled r4 glial cells of the median raphe which show the characteristic radial ventriculo-pial morphology are observed in our sagittally sectioned material.

Midsagittal sections show that the intensely labelled median glial raphe cells belonging to r4 encompass the major part of the pontine bulge in a fan-like median sagittal expansion (Figs. 3j, 4j, 5w–y, 7t). A smaller rostral sector of the pontine bulge whose raphe remains largely unlabelled probably corresponds to r3 (e.g. Fig. 7s, t). Interestingly, there usually appear some labelled median radial glial cells outside of r4, either in r3 or r5. This was not observed in other parts of the neural wall and may be due to the reported absence of interrhombomeric clonal restriction boundaries across the floor plate (Fraser et al. 1990); unfortunately, this cannot be verified in our material, since our labelling is polyclonal.

### Labelled fibre tracts

**Fig. 17:**
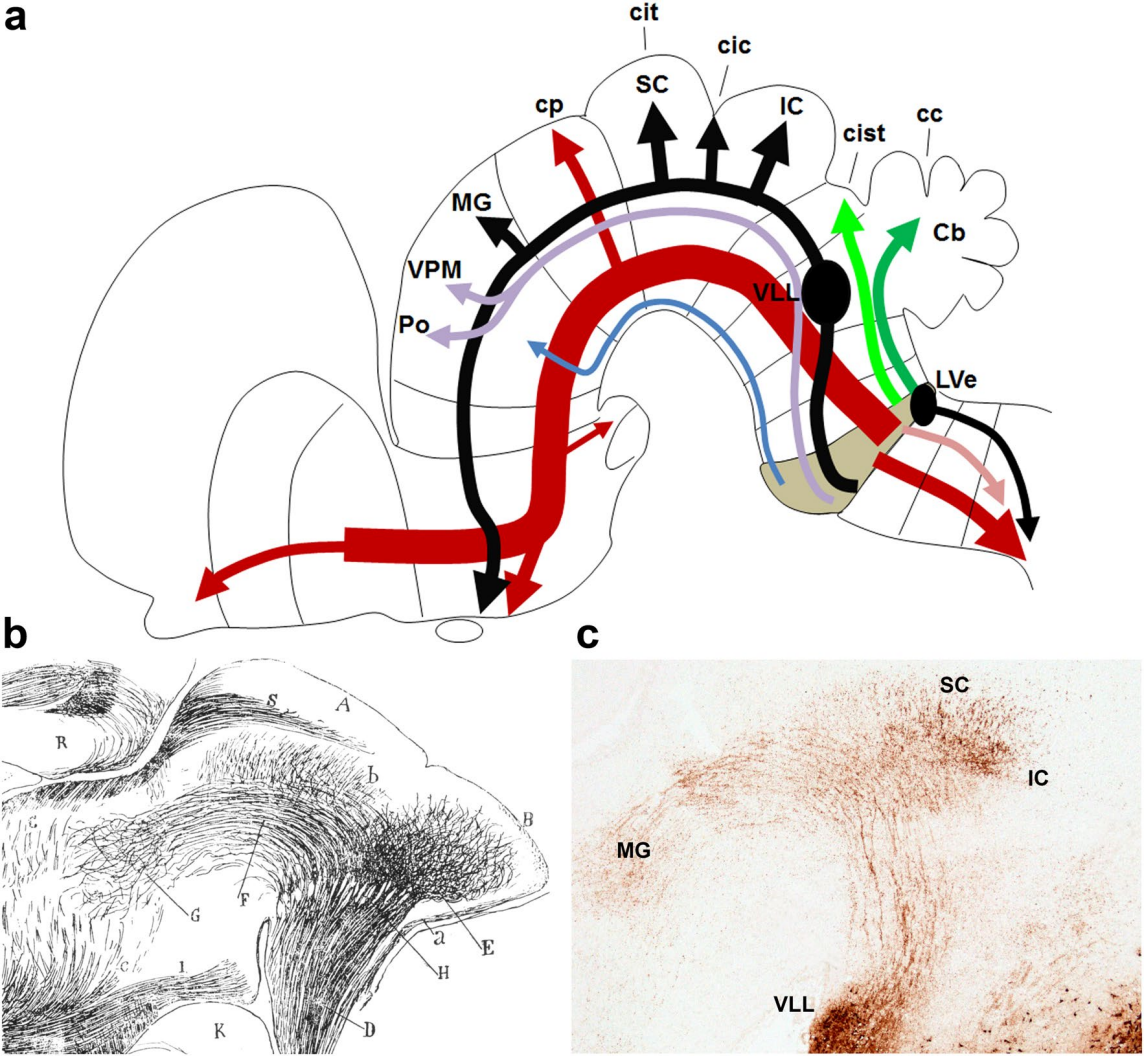
**a** Schematic representation of the ascending and descending r4-derived tracts. Ascending r4-derived tracts include the lateral lemniscus with multiple midbrain and diencephalic terminals (*black*), the medial tegmental tract entering the medial forebrain bundle (*red*), the superficial ascending tract (*blue*), the trigeminothalamic tract (*violet*) and the trigeminal and vestibular cerebellopetal tracts (*light green* and *dark green*, respectively). Descending r4-derived tracts include the medial spinopetal tract (including medial reticulospinal and medial vestibulospinal fibres) (*red*), the lateral trigeminal oro-spinal tract (*pink*) and the lateral vestibulospinal tract (*black*). **b** Ramon y Cajal’s 1911 remarkable drawing of the distribution of the lateral lemniscus with terminals reaching inferior colliculus, superior colliculus and thalamus, in agreement with the present results. **c** A YFP-immunoreacted sagittal section from our material (E18.5; *b1r4-Cre*/*YFP* brain), illustrating a similar view of the lateral lemniscus

A surprising number of tracts appear labelled in our material (Fig. 17). Some of them can be identified confidently, though they present novel aspects, whereas others must be given tentative identities. Clearly, we can provide here only a preliminary descriptive analysis and this subject will need additional experimental work.

#### Lateral lemniscus

As mentioned above in connection with the ventral nucleus of the lateral lemniscus, the tangentially migrating lemniscal neurons are accompanied by labelled growing fibres within the tract, which presumably come from projection neurons in the r4 module of the contralateral cochlear nuclei as well as from the migrating VLL neurons themselves, plus the small r4-derived component of ipsilateral SOC cells (Malmierca and Merchán 2004). These fibres reach the inferior colliculus (deep to its marginal stratum) between E14.5 and E16.5 (Figs. 3u, 4a–c, 5c–g), but do not penetrate radially into its deeper parts yet; this happens between E16.5 and E18.5 (Figs. 7c–h, 9c–f). Surprisingly, already at E14.5 we observed that many of these labelled lemniscal fibres extend through the brachium of the inferior colliculus into the thalamus (Fig. 3l–o) and beyond. In the thalamus, the labelled lateral lemniscal fibres course longitudinally roughly through the area occupied by the medial geniculate nucleus (Figs. 8a–c, 11d–j, 13a–e); the packet of fibres then progressively approaches the brain surface more rostrally (Figs. 4a, b, 5m–o, 6a–d). Rostral to the medial geniculate body (prethalamus), the lemniscal fibres adopt a subpial position ventral to the optic tract, and at E16.5 they extend across the hypothalamus all the way to the supraoptic decussation (Figs. 5p–y, 6g–i). At E18.5 and P8, the lateral lemniscus shows labelled profuse terminal innervation of the inferior colliculus, as well as labelled fibres in the intercollicular commissure; moreover, collaterals from the ascending lateral lemniscus fibres in the brachium of the inferior colliculus enter the deep stratum of the superior colliculus (superficial to the periaqueductal grey), and some of them extend into the tectal commissure (Figs. 7b–t, 8, 9a–f, 11e–q).

#### Medial tegmental tract and medial forebrain bundle

Another massive ascending longitudinal tract coming out of r4 forms a thick ascending packet in the medial tegmentum of the upper hindbrain; afterwards, it crosses the midbrain and diencephalic tegmentum in a similar position and apparently contributes within the lateral hypothalamus to the medial forebrain bundle; the tract eventually reaches the telencephalon via the peduncular hypothalamus (Puelles et al. 2012, 2013; Puelles and Rubenstein 2015). We assume that this packet of fibres is a segmental r4 component of a larger tract with other segmental contributors, probably the central tegmental tract (see “Discussion”), though it might include some other components. The earliest appearance of the r4-labelled medial tegmental tract is at E11.5. Its fibres barely reach the isthmus at this stage (Fig. 1h/hʹ, i/iʹ). At E12.5, they extend across the midbrain and diencephalic tegmentum, following the curve of the cephalic flexure (Fig. 2b–d), and they enter next the peduncular domain of the basal hypothalamus at E14.5 (Fig. 3e–h, p–x). At E16.5, some labelled fibres of this tract course longitudinally through the whole basal hypothalamus, reaching the supraoptic decussation, whereas others turn sharply into the medial forebrain bundle as it ascends through the alar peduncular hypothalamus (Figs. 4b–i, 5d–y, 6a–i). These accordingly incorporate into the telencephalic peduncle and penetrate the telencephalon. As the tract passes through the subparaventricular area of the peduncular hypothalamus, some fibres diverge radially, approaching a specific unidentified area of the hypothalamic periventricular stratum possibly coinciding with the preincertal area; collaterals are given also ventrally to the mamillary/retromamillary periventricular area (not shown). Detailed examination at high magnification indicated that most labelled fibres reaching the telencephalon through this pathway disperse and end within the subpallium (mainly substantia innominata and pallidum). We found only one or two fibres that crossed the striatum and passed beyond the palliosubpallial boundary into the pallium (not shown). Some terminal fibres alternatively extend medially into the anterior commissure and the septum (not shown). At E18.5, the medial tegmental tract displays collaterals that grow into the posterior commissure and nearby areas of the pretectum, and it may contribute as well to the superior colliculus and the tectal commissure (Figs. 7j–t, 8g–i). The origin of the labelled fibres of the medial tegmental tract is uncertain, but the best candidate seems to be the r4 reticular formation (see “Discussion”).

#### Superficial ascending tract

At E16.5, there appears subpially a small labelled ascending axon bundle lying superficial to the medial tegmental bundle, which might arise from a superficial group of dispersed labelled neurons found just rostral to the pons; this tract could be followed into the midbrain paramedian tegmentum, where it either ends or is incorporated by the medial tegmental tract (Figs. 5l–t, 6k, l).

#### Trigeminothalamic tract

We observed at E16.5 a small packet of labelled fibres that occupy a position intermediate between the lateral lemniscus and the medial tegmental tract in the upper brainstem (Fig. 5g–k). These fibres can be followed at higher magnification into the posterior thalamic nucleus (Po) and the medial part of the ventrobasal complex (VPM) (Figs. 7i–k, 8d–f, 11h–m, 13d–f). We therefore assume that they represent labelled r4 components of one of the trigeminothalamic projection tracts, possibly the ventral trigeminothalamic tract, given the presumed origin of such projections in the oral subnucleus of the trigeminal descending column (De Chazeron et al. 2004; Guy et al. 2005; Veinante et al. 2000; Fig. 14a–e). These fibres probably cross the r4 floor plate (in contrast, the decussation of trigeminothalamic fibres originating in the principal sensory nucleus—the trigeminal lemniscus—apparently occurs across the r2 floor plate, just rostral to the pons, where we do not observe labelled decussated fibres).

#### Cerebellopetal trigeminal and vestibular tracts

We found two separate sets of labelled fibres that enter the cerebellum: one composed of thick, coarse fibres originated in the r4 part of the oral sensory trigeminal subnucleus and the other formed by thin fibres related to the vestibular system. The thicker fibres were observed already at E12.5 (Fig. 2g–i). At E14.5 and E16.5, they occupy an intermediate radial position in the r1 alar plate and seem to stream out of the trigeminal sensory column (Figs. 3u, 4a–c, 5k–w); they approach in an arch the superior cerebellar peduncle at the roof of the cerebellar plate, behind the isthmus, passing through a nuclear aggregate (possibly a cerebellar nucleus, or the parabrachial complex) and penetrating finally the incipient cerebellar commissure (Figs. 3b–h, j, p, 4a–i, 5f–j); this cerebellopetal trajectory recalls that of the indirect spino-cerebellar tract (which carries spinal collateral motor copy signals to the cerebellum), but has a trigeminal source in r4, thus possibly mediating analogous cerebellar input from the oral region.

The thinner fibres were only observed after E16.5, but then they already cross the midline of the cerebellar nodule (Fig. 5f), so their invasion of the cerebellar primordium via the inferior cerebellar peduncle possibly occurs at E15. These fibres can be traced back along a superficial pathway into the vestibular complex and may thus be vestibulocerebellar (or reticulo-cerebellar) in nature (Fig. 5f–w).

#### Medial reticulospinal and vestibulospinal tracts

Fibres descending from r4 into the spinal cord already exit from the medial r4 tegmentum at E11.5 (Figs. 1i–k, 2a–e). At E14.5, they approach the upper limit of the spinal cord (Fig. 3h, i) and at E16.5 they build a massive bundle that descends medially through the medulla ventral to the medial longitudinal tract (Figs. 4c–i, 5q–y, 7f–t, 9e–h) and then adopts a superficial position in the ventral column of the spinal cord (Fig. 5v–y). These descending fibres may originate in reticular and vestibular neurons from r4.

#### Lateral trigeminal oro-spinal tract

We observed this tract from E16.5 onwards. It consists of thin fibres that course in an intermediate (lateral basal) tegmental position, intercalated between the medial reticulospinal and vestibulospinal tracts and the lateral vestibulospinal tract (Figs. 5r–w, 9d–h). These fibres finally arch into the lateral column of the spinal cord (not shown). They may correspond to the descending projections of the oral trigeminal subnucleus to the cervical spinal cord (Devoize et al. 2010).

#### Lateral vestibulospinal tract

These fibres were distinguished at E16.5. They seem to originate from the labelled neurons of the lateral vestibular nucleus in r4 (and from those migrated into r3 and r5) (Di Bonito et al. 2015) and have a separate far lateral descending course into the lateral column of the spinal cord (Figs. 4c, d, 5l–u, 7h–l, 9a, b, d–h).

## Discussion

In this work, we used a rhombomere-specific *Cre-recombinase* mouse line crossed with a floxed YFP reporter line to genetically label rhombomere 4 and its derivatives. Going beyond the preliminary data recorded by Di Bonito et al. (2015) and Di Bonito et al. (2013a, b), we mapped in detail the anatomical fate of tangentially and radially migrated r4-derived neuronal populations from embryogenesis to adult, analysing also the r4-originated fibre tracts.

Quail-chick grafting experiments (Cambronero and Puelles 2000; Marin and Puelles 1995; Wingate and Lumsden 1996) and mouse rhombomere-specific transgenic fate-mapping data (Di Bonito et al. 2013a, 2015; Di Meglio et al. 2013; Farago et al. 2006; Oury et al. 2006; Pasqualetti et al. 2007; present work) have shown the persistence of both overt and cryptic rhombomere-derived territories in the brain wall until adult stages, irrespective of the fact that advancing maturation of the hindbrain eventually hinders their non-experimental visualization.

Such data have corroborated that the hindbrain is organized in longitudinal columns of sensory and motor nuclei, which are subdivided into discrete segmental or neuromeric units. The molecular boundaries of overt and cryptic rhombomeres correlate topographically with the transverse limits of nuclei, or of distinct columnar modular subdivisions, as has been visualized according to co-linear differential expression of *Hox* genes (Cambronero and Puelles 2000; Marin et al. 2008; Marin and Puelles 1995; Tomas-Roca et al. 2016). The classic columns thus have a plurisegmental origin; it is believed that the subtle molecular differences that distinguish segmental modules one from another (a result of anteroposterior patterning) causes the columns to be structurally and functionally heterogeneous lengthwise (cell properties, local circuitry, long-range afferents and projections). The original *Hox* gene code of each neuromeric columnar subdomain, jointly with other AP molecular determinants and differential DV expression of transcription factors, appears to determine the ulterior development of specific neuronal identities or cell arrangements inside the intracolumnar modules. This pattern underlies on the whole the observed heterogeneity of neuronal populations within the DV sensorimotor columns along the AP axis (Di Bonito et al. 2013a, b, 2015; Di Meglio et al. 2013; Marin et al. 2008; Oury et al. 2006; Philippidou and Dasen 2013; Puelles et al. 2013; Tomas-Roca et al. 2016).

In particular, we show here that r4 contributes from dorsal to ventral to a particular neuromeric module of the auditory, vestibular and trigeminal sensory columns, as well as of the intermediate and lateral reticular formation, and it produces likewise specific tangentially migrated cell populations.

### R4-derived auditory system

The fact that r4 contributes both to the posteroventral cochlear nucleus and the overlying rostroventral part of the dorsal cochlear nucleus, while only few labelled cells appear in the anteroventral cochlear nucleus and rostralmost dorsal nucleus, or at the caudal end of the cochlear column, implies that the cochlear column is double, that is, it is truly composed of dorsal and ventral subcolumns, rather than representing unitary cochlear nuclei aligned in a deformed topologically longitudinal series, as has been hitherto assumed (Malmierca and Merchán 2004). Each subcolumn displays its own set of neuromeric modules. This conclusion seems to be of considerable interest for comparative considerations, since homologs of the mammalian dorsal and ventral cochlear nuclei in non-mammals were generally expected to be arranged singly along the rostrocaudal axis, and no satisfactory solution has emerged so far. A small rostral part of the lateral superior olive likewise shows r4-derived neurons, as described in the chick superior olive (Marin and Puelles 1995), though it is not clear that these formations are really homologous. Other r4-derived audition-related neurons migrate rostralwards out of r4, apparently guided by the incipient lateral lemniscus tract, eventually forming the interstitial ventral nucleus of the lateral lemniscus that stretches after its tangential migration across r4, r3 and r2. Its dorsoventral origin within r4 is suggested by the facts that VLL cells share a GABAergic/glycinergic phenotype (e.g. expression of *GlyT2, GAD67, VIAAT*) with neurons in the lateral, ventral and medial nuclei of the trapezoid body, and both VLL and the trapezoid complex derive from an *En1Cre* genetic lineage (Altieri et al. 2015, 2016). The latter is topographically restricted to the basal plate, which indicates that the VLL migration probably has a basal origin. Curiously, there are three conventionally described lemniscal nuclei, namely ventral, intermediate and dorsal ones (VLL, ILL, DLL), but all three seem to have different origins and are molecularly diverse, and their lineal order along the lateral lemniscus is actually caudo-rostral, rather than ventro-dorsal (there is a local bending of the axial dimension at the lower limb of the cephalic flexure).

We previously reported that VLL coming from r4 reaches r1 levels (Di Bonito et al. 2013a), but we now hold that the rostral alar r1 locus within the lateral lemniscus is occupied instead by the *Pax7*-positive dorsal lemniscal nucleus (DLL), thought to originate from alar r1 (*Pax7* is not expressed in the hindbrain basal plate—Allen Developing Mouse Brain Atlas; but is expressed ubiquitously in the ventricular zone of the hindbrain *alar* plate; nevertheless, *Pax7*-positive neurons entering the mantle layer are generated exclusively in r1; see Ju et al. 2004; Lorente-Canovas et al. 2012; Moreno-Bravo et al. 2014). Both DLL and VLL are GABAergic/glycinergic populations, but alar-derived DLL is unlabelled in our material, while basal-derived VLL is YFP positive. The small intermediate lemniscal nucleus or ILL also remains unlabelled in our material; however, it is *Pax7* negative (i.e. is not r1 derived) and expresses glutamatergic markers. Accordingly, it represents a separate lemniscal population whose so far unknown AP origin must be different from those of DLL and VLL, possibly somewhere in r2 or r3; as regards its DV origin, it derives from an *Atoh1*/*Wnt1-*positive alar plate domain (Machold and Fishell 2005; Rose et al. 2009).

The lateral lemniscus fibres reach the deep stratum (central nucleus) of the inferior colliculus and also target the superior colliculus: some fibres decussate through the intercollicular and tectal commissures. It was a surprise to find that many lateral lemniscus fibres extend through the brachium of the inferior colliculus into the primordium of the medial geniculate nucleus in the thalamus and even farther ahead. Remarkably, it turns out that both the lateral lemniscal connection with the superior colliculus and the thalamic connection were illustrated by Ramón y Cajal on the basis of Golgi observations (Ramon Cajal 1911; Fig. 17b). Our results indicate that this ascending branch of the lateral lemniscus extends beyond the medial geniculate body, adopting thereafter a subpial position ventral to the optic tract, and reaches the supraoptic decussation, presumably targeting thereafter the contralateral medial geniculate nucleus.

In the superior olivary complex, r4 forms a small part of LSO and contributes to the dorsal (DPO) and rostral (RPO) periolivary regions, as well as to the lateral and medial olivocochlear efferent neurons (LOC, MOC). The cholinergic LOC and MOC populations originate jointly with facial branchiomotor motoneurons and vestibular efferent neurons in the basal plate of r4. The axons of the vestibular efferent neurons enter the vestibulocochlear nerve together with the olivocochlear fibres.

### R4-derived vestibular system

The r4 module of the vestibular column produces large stellate neurons that in part remain in the lateral vestibular nucleus within r4 and in part invade r3 rostrally and r5 caudally (neighbouring neuromeric portions of the superior and inferior vestibular nuclei, respectively). These large vestibular cells represent an anatomically heterogeneous group of descending vestibular projection neurons. The r4-derived vestibular neurons largely project ipsilaterally to the spinal cord forming the lateral vestibulospinal tract; other neurons project contralaterally via the medial vestibulospinal tract (Di Bonito et al. 2015; Diaz et al. 2003, 1998). Thinner fibres originating likewise in the r4 module of the vestibular complex enter the cerebellum via the vestibulocerebellar tract, which crosses the midline of the cerebellar nodule.

### R4-derived trigeminal system

In the trigeminal column, r4 forms the rostralmost segmental module of the oral subnucleus of the descending trigeminal column; this unit contributes to the lateral trigeminothalamic tract projecting to the posterior and ventral posteromedial thalamic nuclei (De Chazeron et al. 2004; Guy et al. 2005; Veinante et al. 2000). In contrast, the principal sensory nucleus maps to r2 and r3 (Oury et al. 2006) and projects to the thalamus via the ventral (crossed) and dorsal (ipsilateral) trigeminothalamic tracts. Thus, *Hox* collinearity differentiating the rhombomeric origins of Pr5 in r2 (*Hoxa2* ^*low*^)-r3 (*Hoxa2* ^*high*^) and Sp5O in r4 (*Hoxb1*) is consistent with the differential hodologic properties of their distinct trigeminothalamic pathways (Di Bonito et al. 2013b; Pouchelon et al. 2012). In addition, thick labelled fibres stream out of the r4 module of the trigeminal sensory column as part of the trigeminocerebellar tract, which decussates within the intracerebellar commissure. Watson and Switzer (1978) and Fu et al. (2011) described rhombic lip-derived precerebellar neurons in the Pr5, but deduced an origin caudal to r4, which excludes a correlation with our data; however, it may be speculated that the coarse labelled trigeminocerebellar projections we see arising from the oral trigeminal subnucleus may come from similar precerebellar rhombic lip cells originated within r4 that migrate into Sp5O. The lateral trigeminal oro-spinal tract described by Devoize et al. (2010) also contains fibres originated in r4.

### R4-derived reticular system

The hindbrain reticular formation generally consists of medial, intermediate and lateral domains (Jones 1995); the medial large-cell component occupies the basal plate, whereas the intermediate medium-size component occupies the ventral rim of the alar plate where tangentially migrated preganglionic parasympathetic, branchiomotor and noradrenergic neurons tend to aggregate (Blessing 1997; see his Figs. 8.3, 8.6; see also Ju et al. 2004). The lateral parvocellular component lies deep to the sensory columns, farther dorsally in the alar plate. The small lateral reticular neurons serve as interneurons for reflex sensorimotor circuitry. The r4 module contributes as expected to the whole local reticular formation, roughly at the level of the caudal pontine reticular nucleus and rostral to the nucleus raphe magnus (ascribed to r5–r6; Alonso et al. 2013); this sector seems to contribute likewise to the shell of the posterodorsal tegmental area and the nucleus subcoeruleus; these entities lie rostrally in r2 (caudal to locus coeruleus in r1; see shell of PDTg in Fig. 14d). Reticular fibres exit caudalwards from the r4 tegmentum forming the medial reticulospinal descending tract, which adopts a superficial position in the ventral spinal cord column. Some r4-derived reticular neurons projecting to the spinal cord translocate into r5 (Di Bonito et al. 2015).

A remarkable finding of the present study was the thick longitudinal tract that ascends through the medial (basal) tegmentum of the upper hindbrain, midbrain and diencephalon. This tract reaches the supraoptic decussation and, separately, the telencephalon via the medial forebrain bundle, coursing ventrodorsally through the peduncular lateral hypothalamus (hypothalamic prosomere 1 of Puelles 2013; Puelles et al. 2012, 2013; Puelles and Rubenstein 2015). As this tract transits through the midbrain and caudal diencephalon, it emits branches into the superior colliculus and tectal commissure, as well as into the posterior commissure and nearby areas of the pretectum, but was not seen to penetrate the thalamus. The r4-derived fibres that ascend through the medial forebrain bundle seem to end mainly at the basal telencephalon (subpallium).

The observed tract probably represents just the r4 segmental (caudal pontine) component of a larger entity to which other rhombomeres may also contribute (Jones 1995). Its remarkable volume and sparse *en route* terminations is somewhat surprising. The precise neuronal origin of these ascending r4 fibres is not distinguishable in our material. The most distinct possibility is that the ascending r4 medial tegmental tract originates from the local medial large-celled component of the reticular formation, known to send fibres through the diencephalic tegmentum into the medial forebrain bundle, connecting there with hypothalamo-cortical neurons (Jones and Yang 1985; Saper 1985; Vertes and Martin 1988; Vertes et al. 1986). As a potential alerting system, this pathway is unusual in seemingly bypassing the intralaminar thalamus (though more detailed observations might detect such connections).

Alternatively, or in addition, the observed terminal projections into superior colliculus, pretectum and mamillary/retromamillary areas might be consistent with an r4-derived vestibular input to circuitry generating head-direction coding properties in neurons at these target sites, extending as well into the basal telencephalon. It is generally assumed that this functional specialization requires visual, head proprioception and vestibular input, which might come in part from r4. This conjecture unfortunately lacks presently a strong basis, since relevant connections into the head-direction system have been associated so far to the mutually connected lateral mamillary nucleus and dorsal tegmental nucleus (in r1) and to the afferents of the latter from the nucleus prepositus hypoglossi and the medial vestibular nucleus, which convey horizontal canal input (e.g. Bassett and Taube 2005; Hopkins 2005). However, the nucleus prepositus hypoglossi, jointly with the medial vestibular nucleus, lies intercalated between the abducens nucleus (r5) and the rostral end of the hypoglossal nucleus (r8), and therefore would not be expected to be labelled in our material. It remains nevertheless possible that other than horizontal head velocity vestibular input—e.g. head pitch, or eye position—is conveyed to the head-direction system by other parts of the vestibular column, part of which may lie in r4.

### R4-derived functional subcircuits in sensorimotor systems

In the auditory system, the r4-derived part of the CN and VLL contribute to the main sound transmission pathway (Di Bonito et al. 2013a). The parallel pathway for sound localization in space instead involves the r2/r3-derived parts of the cochlear nucleus and the superior olivary complex (SOC), which is mostly derived from r5 (Di Bonito et al. 2013a; Farago et al. 2006; Maricich et al. 2009; Marin and Puelles 1995). Moreover, centrifugal acoustic cell populations originated in the r4 basal plate (FBM and MOC) represent two distinct auditory sensorimotor feedback subcircuits (MEM and MOC reflexes) essential to protect the cochlea from acoustic overstimulation. Innervation of auditory receptor cells by MOC is also probably important for their survival, as well as for their function in the cochlear amplification process (Di Bonito et al. 2013a).

In the vestibular system, r4 derivatives contribute to the lateral and medial vestibulospinal pathways that regulate trunk and limb musculature that counteracts perturbations of body position (Di Bonito et al. 2015), as well as to the vestibular efferent neurons. Apart from postural, vestibulo-ocular and optokinetic reflexes, segmental components of the vestibular column might also be involved in the generation of head-direction properties.

Facial somatosensory inputs are variously transmitted through the heterogeneous trigeminal column to the brainstem and thalamus (Pouchelon et al. 2012). Along the AP axis, four distinct trigeminal pathways have been reported to convey input from the face to somatosensory cortex, associated with different parts of the principal (Pr5) and spinal (Sp5) nuclei. Crossed and ipsilateral trigeminal lemniscal pathways are associated to r2- and r3-derived parts of Pr5 conveying proprioceptive and tactile facial stimuli; other crossed trigeminothalamic pathways arise from the Sp5O and the Sp5I subnuclei (Oury et al. 2006; Pouchelon et al. 2012). We found that some Sp5O neurons projecting to the VPM and Po derive from r4, but we ignore whether additional such elements arise likewise more caudally in r5 (Di Bonito et al. 2013b). The Sp5O is involved in the processing of somatosensory input and nociceptive stimuli from the orofacial region (Dallel et al. 1999, 1990; Raboisson et al. 1995) and contains interneurons taking part in various reflex activities (Abrahams and Richmond 1977; Olsson et al. 1986). The Sp5O has also been suggested to be involved in the coordination of head–neck movements and related modulation of incoming sensory information from the cervical spinal cord (Devoize et al. 2010). Each trigeminal subnucleus apparently has distinct cytoarchitecture, connectivity and function, a feature that seems correlated to their differential rhombomeric origins and the underlying molecular identities.

In conclusion, different rhombomeres contribute via multiple distinct modular units to different subcircuits with specific functions within distinct sensorimotor systems. Indeed, r4-derived nuclei and fibre tracts contribute to rhombomere-specific functional subcircuits in the auditory, trigeminal and vestibular sensorimotor systems. The r4-derived descending reticular tracts attend postural reflexes, while the corresponding ascending reticular circuitry needs to be better characterized, since it seems relevant at least for the ascending reticulo-hypothalamic alerting system, but possibly also for the head-direction system, jointly with other vestibular and reticular modular elements.

## Abbreviations

7N: Facial nucleus
7n: Facial nerve or root
8n: Vestibulocochlear nerve
ac: Anterior commissure
AVCN: Anteroventral cochlear nucleus
APTD: Anterior pretectal nucleus, dorsal part
APTV: Anterior pretectal nucleus, ventral part
Cb: Cerebellum
cc: Cerebellar commissure
cic: Intercollicular commissure
cist: Isthmic commissure
cit: Tectal commissure
cp: Cerebellar peduncle
CPO: Caudal periolivary nucleus
DCN: Dorsal cochlear nucleus
DMTg: Dorsomedial tegmental nucleus
DPO: Dorsal periolivary area
DLG: Dorsal lateral geniculate nucleus
DLL: Dorsal nucleus of the lateral lemniscus
FBM: Facial branchiomotor neuron
IC: Inferior colliculus
IEE: Inner efferent neuron
ILL: Intermediate nucleus of the lateral lemniscus
IRt: Intermediate reticular nucleus
IVe: Inferior vestibular nucleus
ll: Lateral lemniscus
LOC: Lateral olivocochlear neurons
LNTB: Lateral nucleus of the trapezoid body
LSO: Lateral superior olive
LVe: Lateral vestibular nucleus
LVPO: Lateroventral periolivary nucleus
MG: Medial geniculate nucleus
MGD: Medial geniculate nucleus, dorsal part
MGM: Medial geniculate nucleus, medial part
MGV: Medial geniculate nucleus, ventral part
Mo5: Motor trigeminal nucleus
MOC: Medial olivocochlear neurons
mnsh: Microneuronal shell
MNTB: Medial nucleus of trapezoid body
MSO: Medial superior olive
opt: Optic tract
pc: Posterior commissure
ped: Peduncle
PHy: Peduncular hypothalamus
Po: Posterior thalamic nucleus
Pn: Pontine grey nucleus
PnC: Pontine reticular nucleus, caudal part
PnO: Pontine reticular nucleus, oral part
PDTg: Posterodorsal tegmental area
Pr5: Principal trigeminal nucleus
PT: Pretectum
PTh: Prethalamus
PVCN: Posteroventral cochlear nucleus
VCN: Ventral cochlear nucleus
r: Rhombomere
RPO: Rostral periolivary region
RtTg: Reticulotegmental nucleus, central part
RtTgP: Reticulotegmental nucleus, pericentral part
Sp5I: Spinal trigeminal nucleus, interpolar part
Sp5O: Spinal trigeminal nucleus, oral part
Sp5ODM: Spinal trigeminal nucleus, dorsomedial oral part
Sp5OVL: Spinal trigeminal nucleus, ventrolateral oral part
SOC: Superior olivary complex
STh: Subthalamic nucleus
SVe: Superior vestibular nucleus
SubC: Subcoeruleus nucleus
SubCD: Subcoeruleus nucleus, dorsal part
SubCV: Subcoeruleus nucleus, ventral part
Th: Thalamus
Ve: Vestibular nucleus
VEN: Vestibular efferent neurons
VLL: Ventral nucleus of the lateral lemniscus
VNTB: Ventral nucleus of the trapezoid body
VPM: Ventral posteromedial nucleus
ZID: Zona incerta, dorsal part
ZIV: Zona incerta, ventral part
